# Whole Exome Re-Sequencing Implicates *CCDC38* and Cilia Structure and Function in Resistance to Smoking Related Airflow Obstruction

**DOI:** 10.1371/journal.pgen.1004314

**Published:** 2014-05-01

**Authors:** Louise V. Wain, Ian Sayers, María Soler Artigas, Michael A. Portelli, Eleftheria Zeggini, Ma'en Obeidat, Don D. Sin, Yohan Bossé, David Nickle, Corry-Anke Brandsma, Anders Malarstig, Ciara Vangjeli, Scott A. Jelinsky, Sally John, Iain Kilty, Tricia McKeever, Nick R. G. Shrine, James P. Cook, Shrina Patel, Tim D. Spector, Edward J. Hollox, Ian P. Hall, Martin D. Tobin

**Affiliations:** 1 University of Leicester, Department of Health Sciences, Leicester, United Kingdom; 2 Division of Respiratory Medicine, University of Nottingham, Queen's Medical Centre, Nottingham, United Kingdom; 3 Wellcome Trust Sanger Institute, Hinxton, Cambridge, United Kingdom; 4 University of British Columbia Centre for Heart Lung Innovation, St. Paul's Hospital, Vancouver, Canada; 5 Institut universitaire de cardiologie et de pneumologie de Québec, Department of Molecular Medicine, Laval University, Québec, Canada; 6 Merck Research Laboratories, Boston, Massachusetts, United States of America; 7 Merck, Rahway, New Jersey, United States of America; 8 University of Groningen, University Medical Center Groningen, Department of Pathology and Medical Biology, GRIAC Research Institute, Groningen, The Netherlands; 9 Pfizer Worldwide R&D, Cambridge, United Kingdom; 10 Pfizer Worldwide R&D, Cambridge, Massachusetts, United States of America; 11 School of Community Health Sciences, University of Nottingham, Nottingham, United Kingdom; 12 Department of Twin Research and Genetic Epidemiology, King's College London, London, United Kingdom; 13 University of Leicester, Department of Genetics, Leicester, United Kingdom; 14 National Institute for Health Research (NIHR) Leicester Respiratory Biomedical Research Unit, Glenfield Hospital, Leicester, United Kingdom; Georgia Institute of Technology, United States of America

## Abstract

Chronic obstructive pulmonary disease (COPD) is a leading cause of global morbidity and mortality and, whilst smoking remains the single most important risk factor, COPD risk is heritable. Of 26 independent genomic regions showing association with lung function in genome-wide association studies, eleven have been reported to show association with airflow obstruction. Although the main risk factor for COPD is smoking, some individuals are observed to have a high forced expired volume in 1 second (FEV_1_) despite many years of heavy smoking. We hypothesised that these “resistant smokers” may harbour variants which protect against lung function decline caused by smoking and provide insight into the genetic determinants of lung health. We undertook whole exome re-sequencing of 100 heavy smokers who had healthy lung function given their age, sex, height and smoking history and applied three complementary approaches to explore the genetic architecture of smoking resistance. Firstly, we identified novel functional variants in the “resistant smokers” and looked for enrichment of these novel variants within biological pathways. Secondly, we undertook association testing of all exonic variants individually with two independent control sets. Thirdly, we undertook gene-based association testing of all exonic variants. Our strongest signal of association with smoking resistance for a non-synonymous SNP was for rs10859974 (P = 2.34×10^−4^) in *CCDC38*, a gene which has previously been reported to show association with FEV_1_/FVC, and we demonstrate moderate expression of CCDC38 in bronchial epithelial cells. We identified an enrichment of novel putatively functional variants in genes related to cilia structure and function in resistant smokers. Ciliary function abnormalities are known to be associated with both smoking and reduced mucociliary clearance in patients with COPD. We suggest that genetic influences on the development or function of cilia in the bronchial epithelium may affect growth of cilia or the extent of damage caused by tobacco smoke.

## Author Summary

Very large genome-wide association studies in general population cohorts have successfully identified at least 26 genes or gene regions associated with lung function and a number of these also show association with chronic obstructive pulmonary disease (COPD). However, these findings explain a small proportion of the heritability of lung function. Although the main risk factor for COPD is smoking, some individuals have normal or good lung function despite many years of heavy smoking. We hypothesised that studying these individuals might tell us more about the genetics of lung health. Re-sequencing of exomes, where all of the variation in the protein-coding portion of the genome can be measured, is a recent approach for the study of low frequency and rare variants. We undertook re-sequencing of the exomes of “resistant smokers” and used publicly available exome data for comparisons. Our findings implicate *CCDC38*, a gene which has previously shown association with lung function in the general population, and genes involved in cilia structure and lung function as having a role in resistance to smoking.

## Introduction

Chronic obstructive pulmonary disease (COPD) is a leading cause of global morbidity and mortality [1] and whilst smoking remains the single most important risk factor, it is also clear that COPD risk is heritable [2]. The genetics underlying COPD are still not fully understood although genome-wide association studies have identified 26 genomic regions showing robust association with lung function [3]–[6] and, of these, 11 have also now shown association with airflow obstruction [7]–[9]. However, the proportion of the variance accounted for by the 26 common genetic variants representing these regions remains modest (∼7.5% for the ratio of forced expired volume in 1 second (FEV_1_) to forced vital capacity (FVC)) [5].

Although over a quarter of the population with a significant smoking history go on to develop COPD [10], some individuals are observed to have preserved lung function as measured by a normal or high FEV_1_ despite many years of heavy smoking. We hypothesised that these “resistant smokers” may harbour rare variants with large effect sizes which protect against lung function decline caused by smoking. Identification of these variants, and the genes that harbour them, could provide further insight into the mechanisms underlying airflow obstruction.

**Figure 1:**
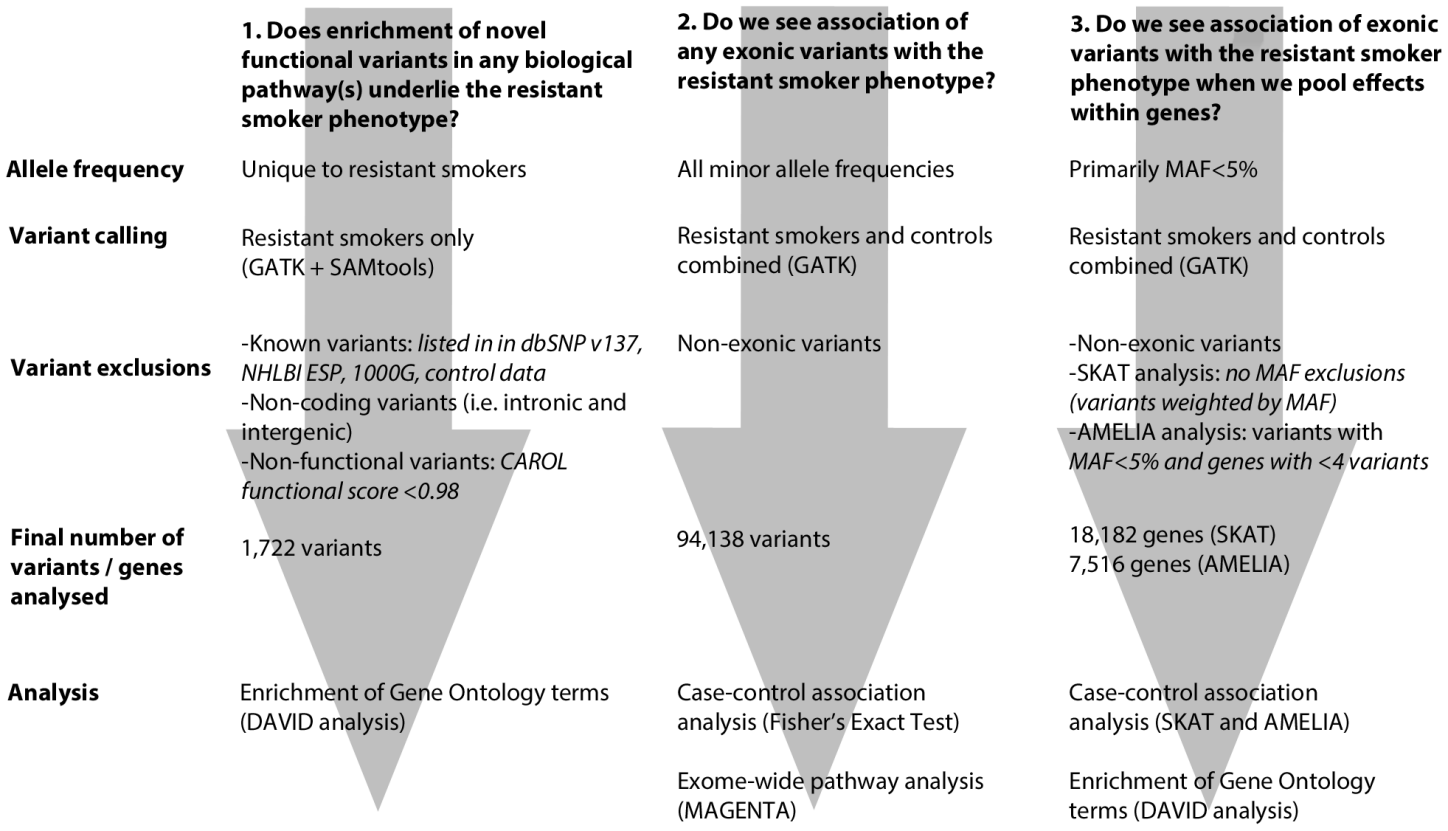
Flowchart describing each of the 3 main analytical pathways used to explore the genetic architecture of smoking resistance. Analyses are described in full in the methods. MAF: minor allele frequency.

We undertook whole exome re-sequencing of 100 heavy smokers (>20 pack years of smoking) who had healthy lung function when age, sex, height and amount smoked were taken into account. We employed 3 complementary approaches to investigate the genetic architecture of the resistant smoker genotype (Figure 1). Firstly, we screened these 100 “resistant smokers” for novel rare variants (i.e. not previously identified and deposited in a public database) with a putatively functional effect on protein product and tested for enrichment of these novel variants in functionally related genes and pathways. Secondly, using a comparision with two independent control sets with exome re-sequencing data, we looked for signals of association with the resistant smoker phenotype for individual variants (including variants of all minor allele frequencies). Thirdly, we looked for association of the resistant smoker phenotype with the combined effects of multiple rare and common variants within genes.

We found the strongest evidence of association with resistance to smoking for a non-synonymous variant in *CCDC38*, a gene encoding a coiled-coil domain protein with a role in motor activity, previously identified as showing an association with lung function. We also show evidence of cytoplasmic expression of *CCDC38* in bronchial columnar epithelial cells. In addition, we found evidence for an enrichment of novel rare functional variants in resistant smokers in gene pathways related to cilia structure and function. Given that abnormalities of ciliary function are already known to play a role in reduced mucociliary clearance in COPD sufferers and smokers, these data suggest that genetic factors may play a significant role in determining the ciliary response of the airway to inhaled tobacco smoke.

## Results

### Sample characteristics

100 individuals from the Gedling [11], [12] and Nottingham Smokers cohorts with good lung function (FEV_1_/FVC>0.7 and % predicted FEV_1_>80%) when age, sex, height and smoking history (>20 pack years) were taken into account were selected as “resistant smoker” cases. Characteristics of the 100 resistant smoker case samples are shown in Table S1A and Figure S1. Exome re-sequencing and alignment was undertaken as described in the methods.

Two independent control sets were used; the robustness of findings using the primary control set (n = 166) were further assessed using a secondary control set (n = 230).

### Identification of novel putatively functional variants in cases

We searched for novel variants among the resistant smokers, i.e. genetic variants which were not observed in either control set and which were not documented in public databases. Bioinformatic tools allow for scoring of likely functional impact, including whether a variant is likely to be “deleterious”; here we use the term “putatively functional” since some variants which have a deleterious effect on the function of a given gene may result in a protective phenotype. A total of 24,098 variants which were not already in databases of known variants or within segmental duplications were identified with high confidence using two independent calling algorithms. A total of 6587 coding SNPs were scored using CAROL (including non-synonymous, loss/gain of stop codon, synonymous and splice site/UTR variants) and 1722 were predicted as being putatively functional (CAROL score>0.98) and were within 1533 genes. 16 of these 1533 genes each contained three novel putatively functional variants (Table S2) (no gene contained more than three such variants). *GBF1* contained three novel putatively functional variants of which one, chr10:104117872, was identified in two case samples. A further 157 genes each contained 2 novel putatively functional variants and the remaining 1360 genes contained one novel putatively functional variant.

In the resistant smokers, there was no overall enrichment or depletion of novel putatively functional variants among the 26 regions reported to be associated with lung function [5], (16 were observed, the same number would have been predicted based on the sequence length of exons) and no novel putatively functional variants were identified within the *CHRNA3/5* region which has been previously associated with smoking [13] and airflow obstruction [9].

Eight of the 1722 novel variants predicted to be putatively functional were identified in >1 case sample. These are listed in Table S3. *ATAD3C* contained a novel putatively functional variant for which six case samples were heterozygous, *SHANK2* contained a novel putatively functional variant for which three cases were heterozygous, and the remaining six genes each contained such a variant for which two cases were heterozygous.

One hundred and ninety two Gene Ontology (GO) terms reached nominal significance for the set of 1533 genes containing novel putatively functional variants in resistant smoker cases. Of these, 22 high level GO terms were significant after Bonferroni correction for multiple testing and are listed in Table 1 [14]. The most significant GO term was the molecular function term “motor activity” which describes molecules involved in catalysis of movement along a polymeric molecule such as a microfilament or microtubule, coupled to the hydrolysis of a nucleoside triphosphate. Other related GO terms also feature amongst the significant signals from this analysis (including “cytoskeleton”, “microtubule motor activity”, “myosin complex”, “axoneme”, “cilium” and “cilium part”) (Table 1 and Table S4).

**Table 1:** Gene Ontology (GO) terms reaching Bonferroni corrected significance for enrichment amongst the 1533 genes harbouring novel putatively functional variants in the resistant smokers, using DAVID.

GO ontology term	# genes overlapping GO term	% of total genes tested overlapping GO term	DAVID EASE P Value	Bonferroni corrected P value
motor activity	39	2.5	1.78×10^−10^	2.08×10^−7^
cell adhesion	105	6.8	3.01×10^−8^	1.02×10^−4^
biological adhesion	105	6.8	3.22×10^−8^	1.09×10^−4^
cytoskeleton	169	11.0	4.30×10^−8^	2.49×10^−5^
adenyl nucleotide binding	191	12.5	7.36×10^−7^	8.59×10^−4^
calcium ion binding	123	8.0	8.65×10^−7^	1.01×10^−3^
ATPase activity	57	3.7	1.14×10^−6^	1.33×10^−3^
purine nucleoside binding	191	12.5	2.08×10^−6^	2.43×10^−3^
nucleoside binding	192	12.5	2.13×10^−6^	2.48×10^−3^
Axoneme	15	1.0	2.44×10^−6^	1.41×10^−3^
ATP binding	178	11.6	2.73×10^−6^	3.18×10^−3^
myosin complex	19	1.2	2.95×10^−6^	1.71×10^−3^
microtubule motor activity	21	1.4	6.05×10^−6^	7.04×10^−3^
adenyl ribonucleotide binding	178	11.6	6.21×10^−6^	7.23×10^−3^
extracellular matrix	53	3.5	1.22×10^−5^	7.06×10^−3^
Cilium	27	1.8	1.41×10^−5^	8.14×10^−3^
proteinaceous extracellular matrix	50	3.3	1.41×10^−5^	8.15×10^−3^
metal ion binding	423	27.6	1.97×10^−5^	2.28×10^−2^
extracellular matrix part	25	1.6	2.20×10^−5^	1.27×10^−2^
cation binding	426	27.8	2.32×10^−5^	2.67×10^−2^
ion binding	431	28.1	2.74×10^−5^	3.15×10^−2^
cilium part	14	0.9	3.29×10^−5^	1.89×10^−2^

### Single-variant association testing

We tested for association of known and novel exonic variants with the resistant smoker phenotype. After exclusion of variants which were missing in >5% of either cases or controls, 269,822 (of which 215,747 were listed in dbSNP137) variants remained. Of the 269,822 variants, 94,138 were exonic and included in further analyses. Similar distributions of variants across the minor allele frequency spectrum were observed for the cases, primary, and secondary controls (results not shown).

**Figure 2:**
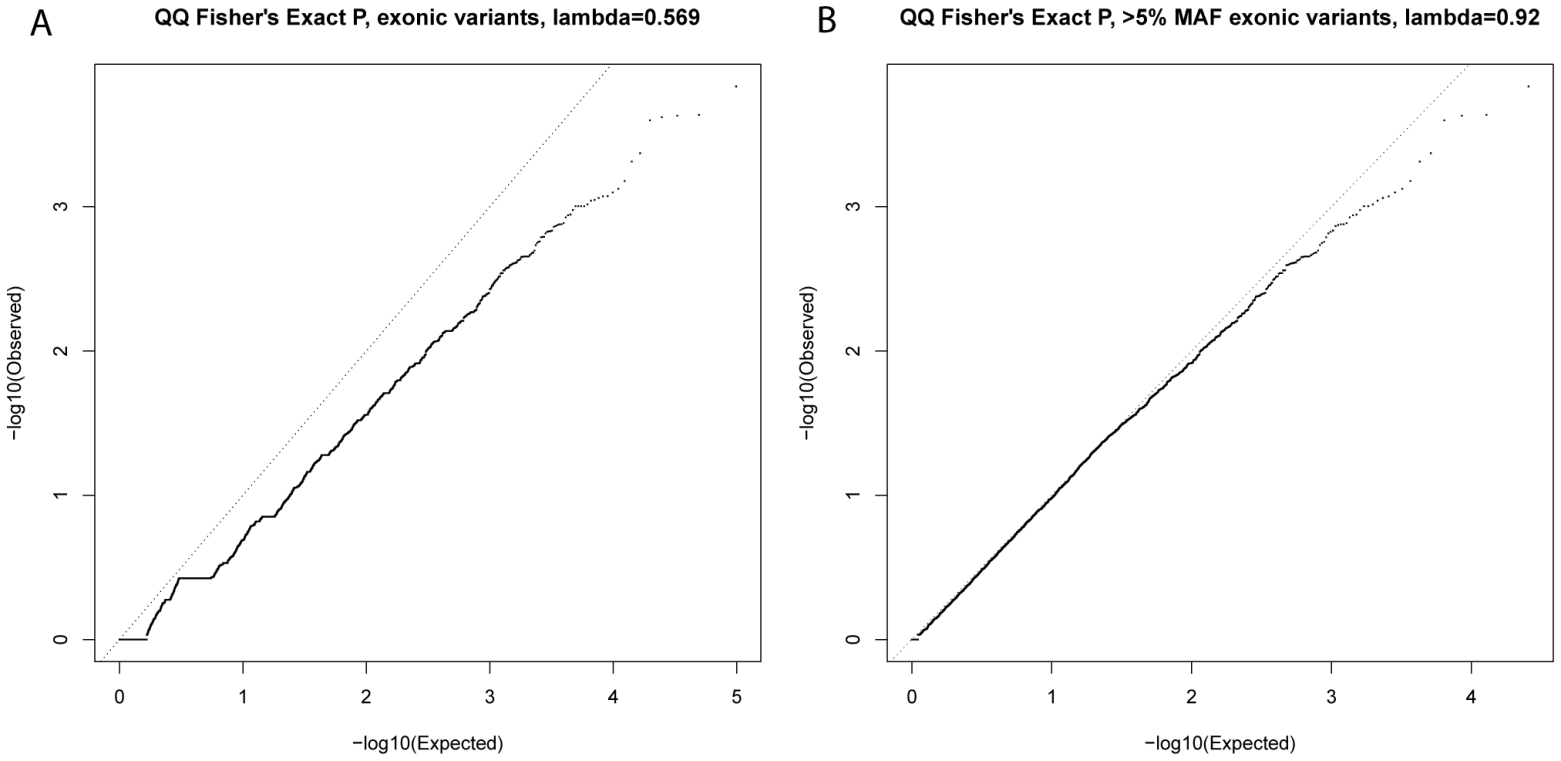
QQ plots of single-variant association test statistics for all exonic variants (A) and for exonic variants with MAF>5% (B).

After testing for association with resistant smoker status using primary controls, no SNPs reached genome-wide significance (P<5×10^−7^, based on Bonferroni correction for 94,138 tests). Substantial under-inflation of the test statistics was seen (lambda = 0.6) (Figure 2A), possibly due to the large number of rare variants (lambda = 0.92 if only variants with MAF>5% [n = 25,646] were considered, Figure 2B). Twenty exonic SNPs showed nominal evidence of association with P<10^−3^ (Table 2).

**Table 2:** Single variant association results for 20 top exonic SNPs.

SNP	Chr	Position (b37)	A1	A2	Resistant smoker cases MAF	Primary control MAF	1000G CEU MAF	Odds Ratio	Fisher P*	SNP type	gene	Gene description
rs1287467	3	15311325	A	G	0.235	0.395	0.342	0.47	1.47×10^−4^	synonymous	*SH3BP5*	SH3-domain binding protein 5 (BTK-associated)
rs2303296	2	24432839	G	A	0.170	0.313	0.292	0.45	2.31×10^−4^	synonymous	*ITSN2*	intersectin 2
rs10859974	12	96288860	C	T	0.255	0.127	0.125	2.36	2.34×10^−4^	nonsynonymous	*CCDC38*	coiled-coil domain containing 38 (CCDC38), mRNA.
rs4850	16	21976762	A	G	0.095	0.021	0.042	4.87	2.40×10^−4^	nonsynonymous	*UQCRC2*	ubiquinol-cytochrome c reductase core protein II, nuclear gene encoding mitochondrial protein
rs1566290	17	71239087	T	G	0.295	0.455	0.383	0.50	2.52×10^−4^	synonymous	*C17orf80*	chromosome 17 open reading frame 80
rs35853276	2	174055646	C	T	0.040	0.133	0.125	0.27	4.26×10^−4^	synonymous	*ZAK*	sterile alpha motif and leucine zipper containing kinase AZK
rs13184586	5	161119125	C	G	0.485	0.331	0.383	1.90	4.87×10^−4^	synonymous	*GABRA6*	gamma-aminobutyric acid (GABA) A receptor, alpha 6
rs2297950	1	203194186	T	C	0.250	0.395	0.350	0.51	6.65×10^−4^	nonsynonymous	*CHIT1*	chitinase 1 (chitotriosidase)
rs7709828	5	94786142	T	C	0.035	0.120	0.142	0.26	7.51×10^−4^	UTR3	*FAM81B*	family with sequence similarity 81, member B
rs1046515	7	140394587	T	C	0.030	0.108	0.075	0.25	7.98×10^−4^	nonsynonymous	*ADCK2*	aarF domain containing kinase 2
rs17010021	2	74761539	A	T	0.000	0.048	0.033	-	8.46×10^−4^	nonsynonymous	*LOXL3*	lysyl oxidase-like 3
rs2878	7	102953621	G	A	0.060	0.157	0.167	0.34	8.49×10^−4^	UTR3	*PMPCB*	peptidase (mitochondrial processing) beta, nuclear gene encoding mitochondrial protein,
rs34350265	9	116973273	T	C	0.115	0.036	0.058	3.47	8.71×10^−4^	synonymous	*COL27A1*	collagen, type XXVII, alpha 1
rs111336032	17	80017898	A	G	0.045	0.003	0.025	15.6	8.98×10^−4^	nonsynonymous	*DUS1L*	dihydrouridine synthase 1-like (S. cerevisiae)
rs6979^1^	16	67691668	G	A	0.575	0.425	0.458	1.83	9.09×10^−4^	nonsynonymous	*ACD*	adrenocortical dysplasia homolog (mouse)
rs2295879	10	123996976	A	G	0.395	0.256	0.358	1.90	9.65×10^−4^	nonsynonymous	*TACC2*	transforming, acidic coiled-coil containing protein 2
rs2427808	1	158577167	T	A	0.170	0.075	0.133	2.52	9.92×10^−4^	synonymous	*OR10Z1*	olfactory receptor, family 10, subfamily Z, member 1
rs17680262^2^	12	110354536	T	C	0.170	0.075	0.075	2.52	9.92×10^−4^	UTR3	*TCHP*	trichoplein, keratin filament binding
rs35464006	17	37840860	C	G	0.035	0.000	NA	-	9.93×10^−4^	nonsynonymous	*PGAP3*	post-GPI attachment to proteins 3
rs116948895	9	84609249	T	A	0.035	0.000	0.008	-	9.93×10^−4^	synonymous	*SPATA3D1*	family with sequence similarity 75, member D1

MAF = minor allele frequency.

*Fisher's Exact test of case and primary control allele counts.

1 Borderline evidence for association with airflow obstruction (P = 0.052 in ever-smokers) [9].

2 Weak evidence of association with airflow obstruction (P = 0.04) [9].

**Figure 3:**
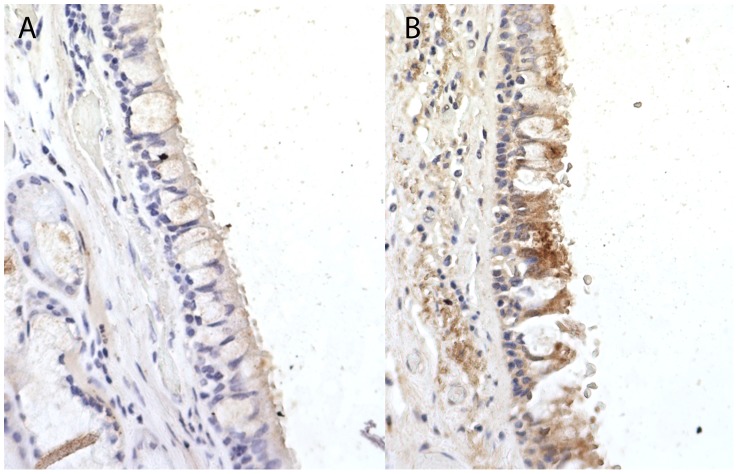
Immunohistochemistry staining for Coiled-Coil Domain-Containing Protein 38 (CCDC38) in a normal human bronchial epithelium at ×40 magnification. When compared to the unstained isotype control (Rabbit IgG) (A), staining identifies moderate cytoplasmic expression of CCDC38 particularly in the columnar epithelial cells and the bronchial smooth muscle layer (B). Image representative of three independent staining procedures.

The strongest signal from a non-synonymous SNP was within a region previously identified as being associated with lung function [5]. The non-synonymous SNP in *CCDC38* (rs10859974, OR = 2.36, P = 2.34×10^−4^) is 17.43 kb away from, but statistically independent of, rs1036429 (intronic, r^2^ = 0.064) which has previously shown genome-wide significant association with FEV_1_/FVC [5]. SNP rs10859974 itself has shown weak evidence of association with FEV_1_/FVC (P = 0.032) [5]. This SNP is predicted to cause a methionine to valine substitution at protein position 227; the valine allele is predicted to be protective. Investigations into CCDC38 expression in bronchial tissue via immunohistochemistry identified moderate staining of CCDC38 in the cytoplasm of columnar epithelial cells, with weak staining in the sub-epithelial layer (Figure 3). We found no evidence that rs10859974 or any of its proxy SNPs (r^2^>0.3) were lung eQTLs for *CCDC38* itself, although rs11108320 which is intronic in *CCDC38* and in strong LD with rs10859974 (r^2^ = 1) is an eQTL for nearby gene *NTN4* (significant at 10% False Discovery Rate (FDR) threshold). Many additional SNPs located near or within *CCDC38* and *SNRPF* were eQTLs for *NTN4* (Table S5). Nearby *CCDC38* intronic SNPs in weaker LD (r^2^ = 0.3) with rs10859974 were eQTLs for *SNRPF* (Table S5).

The strongest signal of association in the single-variant analysis was from a synonymous SNP, rs1287467, in *SH3BP5* (OR = 0.47, P = 1.47×10^−4^) (Table 2). A SNP downstream of *SH3BP5* (rs1318937, 1000G CEU MAF = 0.108, 16 kb from rs1287467, r^2^ = 0.018) has shown evidence of association with alcohol dependence and alcohol and nicotine co-dependence [15].

Synonymous SNP rs2303296 in *ITSN2* was the second strongest signal of association (OR = 0.45, P = 2.31×10^−4^) and had previously shown weak evidence of association with FEV_1_ (P = 0.02) [5] but was not near to any previously identified genome-wide significant associations with lung function and has not shown evidence of association with COPD [9]. Another SNP in *ITSN2*, rs6707600 (intronic, 1000 G CEU MAF = 0.017, 89 Kb from rs2303296, r^2^ = 0.02), has shown some evidence of association with antipsychotic response in schizophrenia patients [16].

The second strongest signal from a non-synonymous SNP was rs4850 in *UQCRC2* (OR = 4.87, P = 2.4×10^−4^). There were no nearby associations reported with any other trait for this gene.

The third strongest signal from a non-synonymous SNP was rs2297950 (OR = 0.51, P = 6.65×10^−4^) in *CHIT1* which encodes chitinase 1 (Chit1). The chitinase pathway has been implicated in asthma and lung function [17] and lung function decline in COPD patients [18]. Chit1 expression in mice has been shown to be correlated with severity of bleomycin-induced pulmonary fibrosis (with overexpression leading to increased severity and *Chit1* ^−/−^ mice exhibiting reduced pulmonary fibrosis) [19].

A non-synonymous SNP in *LOXL3*, rs17010021, was the only SNP with an association P<10^−3^ regardless of whether the primary or the secondary controls were used (Table S6). This variant had a minor allele frequency of 0.048 and 0.061 in the primary and secondary control sets respectively, but the minor allele was not observed in any of the resistant smoker cases.

Synonymous SNP rs1051730, in *CHRNA3* (15q25.1), has previously shown very strong evidence of association with smoking behaviour (particularly cigarettes per day) [13], [20], [21]. This SNP showed weak evidence of association with the resistant smoker phenotype in our study (P = 0.03 when the secondary control set was used and P = 0.06 when primary control set was used). Association results for SNPs within 500 Kb of rs1051730 are in Table S7.

No nominally significant enrichment of association signals in known pathways was identified in the exome-wide results of the single-variant analysis using MAGENTA [22].

### Gene-based association testing

**Figure 4:**
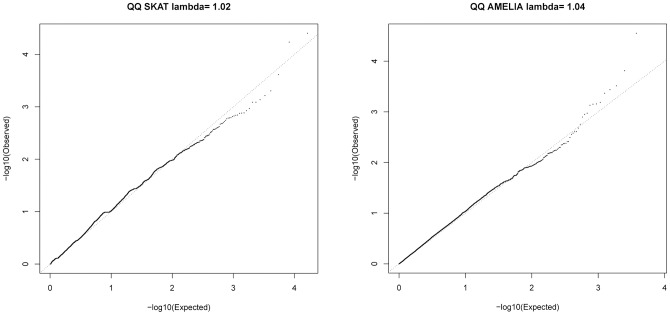
QQ plots for SKAT and AMELIA analyses using primary controls.

SKAT [23] and AMELIA [24] analyses were undertaken to assess whether multiple variants within a gene collectively showed evidence of association; these tests are agnostic to whether a given variant is previously known. Quantile-Quantile plots for SKAT and AMELIA analyses are shown in Figure 4. Genes with nominally significant association (P<10^−3^) for SKAT or AMELIA analysis using the primary controls are shown in Table 3 (results of SKAT and AMELIA analyses using the secondary controls are shown in Table S8). No genes showed significant association after Bonferroni correction for multiple testing (P<0.05/18000 = 2.8×10^−6^) for either analysis (Table 3 and Table S8). Since the genes are likely to be correlated (through LD structure or overlapping reading frames), SKAT provides a resampling function to control Family Wise Error Rate (FWER). No genes were significant after controlling FWER = 0.05. None of the genes in Table 3 and Table S8 were within any of the 26 lung function associated regions [3]–[5], the *CHRNA3/5* smoking-associated region [13] or *SERPINA1* (mutations in which are known to cause alpha-1-antitrypsin deficiency) [25].

**Table 3:** SKAT and AMELIA analysis results using primary controls, ranked by SKAT P value.

	SKAT		AMELIA		Single-Variant	
gene	Variants	SKAT P	<5% MAF	AMELIA P#	Best Fisher's P value	Description
*SPATA3D1*	11	3.62×10^−5^	9	2.80×10^−5^	9.93×10^−4^	family with sequence similarity 75, member D1
*UQCRC2*	4	7.15×10^−5^	0	-	2.40×10^−4^	ubiquinol-cytochrome c reductase core protein II, nuclear gene encoding mitochondrial protein
*UBP1*	8	1.13×10^−4^	5	6.36×10^−3^	1.93×10^−3^	upstream binding protein 1 (LBP-1a)
*TMEM252*	2	4.38×10^−4^	0	-	2.22×10^−3^	chromosome 9 open reading frame 71
*ADCK2*	8	4.76×10^−4^	7	3.66×10^−4^	7.98×10^−4^	aarF domain containing kinase 2
*PGAP3*	2	5.03×10^−4^	0	-	9.93×10^−4^	post-GPI attachment to proteins 3
*CST9L*	3	7.12×10^−4^	0	-	2.22×10^−3^	cystatin 9-like
*UPF2*	11	9.60×10^−4^	8	6.98×10^−4^	7.28×10^−3^	UPF2 regulator of nonsense transcripts homolog (yeast)
*TMX3*	5	2.60×10^−3^	5	3.06×10^−4^	1.96×10^−2^	thioredoxin-related transmembrane protein 3
*LPAL2*	9	3.02×10^−3^	6	6.51×10^−4^	1.34×10^−3^	lipoprotein, Lp(a)-like 2, pseudogene
*FAM193A*	10	3.82×10^−3^	10	1.54×10^−4^	3.26×10^−3^	family with sequence similarity 193, member A
*IMPG2*	8	7.54×10^−3^	5	<1.00×10^−7^	1.80×10^−1^	interphotoreceptor matrix proteoglycan 2
*TCOF1*	17	1.34×10^−2^	12	4.29×10^−4^	1.96×10^−2^	Treacher Collins-Franceschetti syndrome 1

# Only genes with >4 SNPs with MAF<5% were tested by AMELIA.

We also checked overlap between the gene-based association testing and single-variant tests. A signal in *TMEM252* (which showed P<10^−3^ in the SKAT analysis regardless of which control set was used) was driven by rs117451470, a non-synonymous SNP, which had P = 2.2×10^−3^ in the single-variant association analysis (the other SNP in *TMEM252*, a singleton novel synonymous variant, had P = 0.38 in the single-variant analysis). Signals in *UQCRC2* (strongest signal using SKAT), *SPATA3D1*, *PGAP3* and *ADCK2* were also driven by variants with P<10^−3^ in the single-variant analysis. Signals from *TMX3*, *IMPG2* and *TCOF1* were not driven by single-variant signals (all SNPs within these genes had P>0.01 in the single variant analysis). *IMPG2* was the strongest signal from the AMELIA analysis and all 8 SNPs within *IMPG2* had no evidence of association in the single-variant analysis (P> = 0.18).

We tested for enrichment of GO terms within the set of genes showing association with P<0.01 in the SKAT analyses. Ten high level GO terms reached nominal significance (P<0.05) for the set of 150 genes identified using SKAT but none were significant after Bonferroni correction for multiple testing [14].

## Discussion

Understanding why some heavy smokers seem to show resistance to the detrimental effects of cigarette smoke on lung function should provide further insight into the genetics of lung function and COPD. We undertook pathway enrichment, single-variant association testing and gene-based association testing analyses on whole exome re-sequencing data from a set of resistant smokers. Although no individual SNP achieved genome-wide statistical significance (P<5×10^−7^), our strongest association signal for a non-synonymous SNP was in *CCDC38*; a gene which has previously shown strong and robust evidence of association with lung function [5]. The intronic SNP previously shown to be associated with lung function (FEV_1_/FVC) and the non-synonymous SNP showing nominally significant association with the resistant smoker phenotype in this study are located close together (17.4 kb apart) but are not well correlated (the non-synonymous SNP has previously shown nominally significant evidence [P<0.05] of association with FEV_1_/FVC). A conditional analysis of these two SNPs was consistent with no statistical correlation between these signals. Although the function of *CCDC38* is not yet well understood, members of the coiled-coil domain protein family are known to have a role in cell motor activity (e.g. myosin) [26] and cilia assembly [27], [28]. Expression of CCDC38 has been identified in the human bronchi of two subjects, with strong cytoplasmic staining in the epithelium and moderate staining in the airway smooth muscle (Human Protein Atlas [http://www.proteinatlas.org] [29]: ENSG00000165972). We experimentally confirmed these findings using immunohistochemistry on lung sections. We observed moderate cytoplasmic CCDC38 staining in bronchial columnar epithelial cells and some potential airway smooth muscle staining. There is no evidence that SNP rs10859974 is an eQTL for *CCDC38* itself, although proxies for rs10859974 are eQTLs for a nearby downstream gene, *NTN4*, encoding Netrin-4 which may play a role in embryonic lung development [30]. Gene Ontology terms shown to be significantly enriched among the novel putatively functional variants identified only in the resistant smokers also pointed to pathways relating to motor activity and the cytoskeleton, including cilia. Another locus showing association with lung function (1p36.13, [5]) also contains a gene encoding a component of cilia (*CROCC* which encodes rootletin, another coiled-coil domain protein) and *Crocc*-null mice have been shown to have impaired cilia with pathogenic consequences to the airways [31]. The enrichment of genes involved in cilia function amongst the results of our analyses supports the importance of cilia function in lung health. Cilia abnormalities are known to be associated with smoking [32], [33], asthma [34], and play a role in COPD [35] where reduced cilia function leads to reduced mucus clearance of the airways. Improving mucociliary clearance is one of the aims of drug therapy for chronic bronchitis in COPD patients (reviewed in [36]).

Impaired cilia function is known to cause a wide range of diseases (collectively known as ciliopathies) many of which include pulmonary symptoms [37]. Primary Ciliary Dyskinesia (PCD) is a rare genetic disorder where respiratory tract cilia function is impaired leading to reduced (or absent) mucus clearance. Mutations in genes which encode components of the cilia have been found to cause several forms of PCD and include the dynein, axonemal heavy chain encoding genes *DNAH11* [38], [39] and *DNAH5* [40] within which resistant smoker-specific novel putatively functional variants were identified in this study (2 such variants were discovered in *DNAH11*). Whilst PCD affects resistance to infection and results in bronchiectasis, abnormal lung function can manifest early in life and progressive airflow obstruction has been observed in later life, although aggressive treatment may prevent the latter [41]. Retinitis pigmentosa is a feature of several ciliopathies, including some with pulmonary involvement (for example, Alstrom Syndrome). Low frequency variants in *IMPG2* (interphotoreceptor matrix proteoglycan 2) collectively showed strong evidence of association (using AMELIA). Variants in *IMPG2* are associated with a form of retinitis pigmentosa [42]. Another retinitis pigmentosa gene, *RP1*, was amongst the 16 genes containing 3 novel putatively functional variants in the resistant smokers. *RP1* encodes part of the photoreceptor axoneme [43], a central component of cilia.

A recent study identified modulators of ciliogenesis using a high throughput assay of *in vitro* RNA interference of 7,784 genes in human retinal pigmented epithelial cells (htRPE) and identified 36 positive modulators and 13 negative modulators of ciliogenesis [44]. These modulators included many genes which did not encode structural cilia proteins and thus were not obvious candidates for a role in cilia function. None of the genes highlighted by the single-variant or gene-based analyses were confirmed as modulators of ciliogenesis although *ITSN2*, which contained one of the top signals in our single-variant analysis, was included in the screen and showed suggestive evidence of a positive role in ciliogenesis but this was not confirmed in a second screen. Two of the genes found to harbour a novel putatively functional variant in the resistant smokers were identified as positive modulators of ciliogenesis: *GSN* (gelsolin) which is a known cilia gene with a role in actin filament organisation and *AGTPBP1* (ATP/GTP binding protein 1) which has a role in tubulin modification.

Collectively, our data show an enrichment of novel putatively functional variants in genes related to cilia structure and function in resistant smokers. Association between smoking and shorter cilia has been reported [32]. The largest genome-wide association with lung function to date supports the notion that the majority of associated variants, including those associated with COPD risk, affect lung function development rather than decline in lung function in adults [5]. If confirmed in other studies, it would be interesting to assess whether genetic influences on the function of cilia primarily affect growth or whether these affect more directly the extent of damage caused by tobacco smoke.

Very large GWAS have identified up to hundreds of common variants each with a modest effect on a variety of phenotypes. However, collectively, these still only explain a very modest proportion of the additive polygenic variance. It has been widely hypothesised that rare variation may account for some of this missing variance [45]. Commercially available SNP arrays have tended to include mostly variants with minor allele frequencies upwards of 5% and rare variants have not been reliably imputed from these. Re-sequencing approaches provide the most accurate platform for the study of exome-wide and genome-wide rare variation. However, there is increasing evidence that rare variants may not account for the missing heritability for all traits [46]. Our study did not find evidence for any individual rare variants with large effects in any of the known lung function associated loci or elsewhere in the exome (albeit in a modest overall sample size), although we did identify significant enrichment of novel rare variants in sets of genes with known functions in pathways which are known to have a role in lung health.

For the single variant analyses, we used Fisher's Exact Test. Whilst this is an appropriate test to use for small cell counts (for example, where minor allele counts are low), alternatives have been recently proposed including the Firth test, and although the optimal approach in the size of study we undertook is not clear from the comparisons shown to date, the Fisher's Exact Test can be more conservative than the Firth test and this may have had some impact on the power of the study [47]. Methods for the analysis of rare variant data are continuing to evolve.

Although this is the first exome re-sequencing study of resistance to airways obstruction among heavy smokers, our study does have potential limitations. Sample size was limited both by availability of individuals with such an extreme phenotype as that we were able to study, and also by current sequencing costs. We were able to utilise re-sequencing data available to the scientific community as control data and therefore maximise the discovery potential of our resources by re-sequencing to a sufficient sequencing read depth for confident rare variant calling. By doing so, and selecting an extreme phenotype group from our sampling frame, we adopted a suitable design to test whether there was enrichment of rare variants of large effect in resistant smokers. The same limitations also impact on the availability of suitable replication studies. In particular, it would have been desirable to undertake replication to support the statistically significant findings of the pathway analysis. However, in the absence of a suitable replication resource, the prior evidence for the role of cilia in lung health does lend support to our findings. As it becomes possible to sample and re-sequence from very large biobanks it should become possible to circumvent these issues in years to come, particularly if the cost of sequencing falls.

As limited information was available on smoking status among the controls, we did not restrict controls to heavy smokers and there is therefore potential for genetic associations to be driven via an effect on smoking behaviour. Nevertheless, our design is also consistent with the detection of association due to primary effect on airways and previous genome-wide association studies of lung function not fully adjusted for smoking have detected loci associated with lung function and COPD which were not associated with smoking behaviour [4], [5]. We saw only a weak association with variants at the *CHRNA3/CHRNA5* locus (the locus at which variants have shown the strongest effect with smoking behaviour [13], [20], [21]).

Misclassification impacts on power; we would have underestimated the power to detect SNP and gene-based associations if the prevalence of resistance to airways obstruction among heavy smokers was greater than we assumed. In a cross-sectional study of this kind, survivor bias could occur if genetic variants influencing survival were under-represented or over-represented in the resistant smokers, but as the mean age of the resistant smokers was 56.4, any survivor bias, if present, is unlikely to have had a major impact. Finally, although we would expect the allele frequencies of the control sets we used to be representative of a general population control set across the vast majority of the genome, biases could potentially be introduced for any genetic variants related to the ascertainment strategy of the control sets. For the main findings we report in this paper, we also present allele frequencies from a public database (1000 Genomes Project); any such bias does not explain our main findings.

In the first deep whole exome re-sequencing study of the resistant smoker phenotype, we have identified an association signal in a region that has already shown robust association with lung function (*CCDC38*) and demonstrate significant enrichment of novel putatively functional variants in genes related to cilia structure. These findings provide insights into the mechanisms underlying preserved lung function in heavy smokers and may reveal mechanisms shared with COPD aetiology.

## Materials and Methods

### Ethics statement

The Gedling study was approved by the Nottingham City Hospital and Nottingham University Ethics committees (MREC/99/4/01) and written informed consent for genetic study was obtained from participants. The Nottingham Smokers study was approved by Nottingham University Medical School Ethical Committee (GM129901/) and written informed consent for genetic study was obtained from participants. The Edinburgh MR-psychosis sample set was compliant with the UK10K Ethical Governance Framework (http://www.uk10k.org/ethics.html) and no restrictions were placed on the use of the genetic data by the scientific community. For TwinsUK, ethics committee approval was obtained from Guy's and St Thomas' Hospital research ethics committee. Tissue for immunohistochemistry was from Nottingham Health Science Biobank (Nottingham, UK) with the required ethical approval (08/H0407/1).

For lung eQTL datasets: At Laval, lung specimens were collected from patients undergoing lung cancer surgery and stored at the “Institut universitaire de cardiologie et de pneumologie de Québec” (IUCPQ) site of the Respiratory Health Network Tissue Bank of the “Fonds de recherche du Québec – Santé” (www.tissuebank.ca). Written informed consent was obtained from all subjects and the study was approved by the IUCPQ ethics committee. At Groningen, lung specimens were provided by the local tissue bank of the Department of Pathology and the study protocol was consistent with the Research Code of the University Medical Center Groningen and Dutch national ethical and professional guidelines (“Code of conduct; Dutch federation of biomedical scientific societies”; http://www.federa.org). At Vancouver, the lung specimens were provided by the James Hogg Research Center Biobank at St Paul's Hospital and subjects provided written informed consent. The study was approved by the ethics committees at the UBC-Providence Health Care Research Institute Ethics Board.

### Sample selection

100 individuals with prolonged exposure to tobacco smoke and unusually good lung function (resistant smokers) were selected from the Gedling and Nottingham Smokers studies, described below.

The Gedling cohort is a general population sample recruited in Nottingham in 1991 (18 to 70 years of age, n = 2,633) [11] and individuals were then followed-up in 2000 (n = 1346) when blood samples were taken for DNA extraction, and FEV_1_ and FVC were measured using a calibrated dry bellows spirometer (Vitalograph, Buckingham, UK), recording the best of three satisfactory attempts [12].

The Nottingham Smokers cohort is an ongoing collection in Nottingham using the following criteria; European ancestry, >40 years of age and smoking history of >10 pack years (currently n = 538). Lung function measurements (FEV_1_ and FVC) were recorded at enrolment using a MicroLab ML3500 spirometer (Micro Medical Ltd, UK) recording the best of three satisfactory attempts.

Our inclusion criteria was; aged over 40 with more than 20 pack years of smoking and no known history of asthma. A total of 184 samples were eligible for this project after further exclusion of individuals with either FEV_1_, FVC or FEV_1_/FVC less than the Lower Limit Normal (LLN) (based on age, sex and height). We calculated residuals after adjusting % predicted FEV_1_ for pack years of smoking and selected the 100 samples with the highest residuals for exome re-sequencing (Figure S1).

Primary controls were from the Edinburgh MR-psychosis set (n = 166) of the UK10K project (http://www.uk10k.org/) and consisted of subjects with schizophrenia, autism or other psychoses, and with mental retardation. No additional phenotype information was available for the primary controls. The TwinsUK secondary control samples (n = 230) were all unrelated females selected from the high and low ends of the pain sensitivity distribution of 2500 volunteers from TwinsUK [48], [49]. Characteristics of the secondary controls are given in Table S1B (note that phenotype information was only available for a subset of the samples). These secondary controls were not included in the main analyses due to the difference in exome coverage. Further phenotype information was not available for either control sample set.

### Exome sequencing

For the 100 resistant smoker case samples, DNA was extracted from whole blood and the Agilent SureSelect All Exon 50 Mb kit was used for enrichment. The 100 resistant smoker samples were individually indexed and grouped into 20 pools of 5 samples. Each pool was sequenced in one lane (20 sequencing lanes in total) using an Illumina HiSeq2000. Sequences were generated as 100 bp paired-end reads. Exome-wide coverage of 97 out of 100 samples was >20 (Figure S2). Three samples had mean sequence depth coverage <20, of these, one appeared to have had poor enrichment (high number of off-target reads), one had a low overall sequence yield and high number of duplicate reads and one had a high number of duplicate reads (but good sequence yield). To preserve power, and because there was no evidence that the sequence data quality for these samples was lower than for the other samples, these 3 samples were not excluded from further analyses.

A total of 166 exomes from the Edinburgh MR-psychosis study: a subset of the neurodevelopmental disease group of the UK10K project (http://www.uk10k.org/), were used as primary controls. These were enriched using the Agilent SureSelect All Exon 50 Mb kit and sequenced using an Illumina HiSeq2000 to a mean coverage depth of ∼70x (75 bp paired-end reads). The sequencing of the secondary controls from the TwinsUK pain study has been described elsewhere [49]. In brief, raw sequence data was available for 230 exomes which had been enriched using the NimbleGen EZ v2 (44 Mb) array and sequenced on an Illumina HiSeq2000 to a mean depth of coverage of 71x (90 bp paired-end reads).

### Sequence alignment

The sequence alignment of the primary control exomes has been described elsewhere (http://www.uk10k.org/). The 100 resistant smoker case exomes and 230 TwinsUK controls were aligned using BWA v0.6.1 [50] with -q15 for read-trimming. Samtools v0.1.18 [51] was used to convert sort, remove duplicates and index the alignment .bam files. GATK v1.4-30 [52] was used to undertake local realignment around indels and to recalibrate quality scores for all 3 datasets.

### Identification of novel putatively functional case-only variants

In order to identify novel variant calls in the 100 resistant smoker exomes, GATK and SAMtools mpileup were run on a per sample basis for all 100 exomes. Only bases with a base quality score >20 were included. The variants called were then compared with dbSNP137, 1000 Genomes Project (1000G) and NHLBI Exome Sequencing Project calls and all known variants were excluded in order to identify novel rare variants which were unique to the 100 resistant smoker exomes. The novel GATK variant calls were then excluded if they had a Phred scaled probability (QUAL) score <30, quality by unfiltered depth (non-REF) (QD) <5, largest contiguous homopolymer run of the variant allele in either direction >5, strand bias >−0.1 or Phred-scaled P value using Fisher's Exact Test to detect strand bias >60. The novel SAMtools mpileup variants were excluded if they had a QUAL<30, mapping quality <25 or genotype quality <25. Variants called at sites with a depth of coverage less than 4 or greater than 2000 were also excluded. The intersect of variants which were identified and passed filtering using both GATK and SAMtools mpileup was taken forward for further analysis.

CAROL (http://www.sanger.ac.uk/resources/software/carol/) was used to predict the consequence of all coding variants. This method combines the results of the functional scoring tools SIFT and Polyphen2. SNPs were predicted as being putatively functional if they had CAROL score>0.98. Amino acid changes were predicted using ENSEMBL.

### Variant calling and QC across cases and controls

For each comparison (resistant smoker cases vs. primary controls and resistant smoker cases vs. secondary controls), variant calling was undertaken across cases and controls together using the GATK v1.5-20 Unified Genotyper. Only bases with a base quality score >20 were included. Coverage was down-sampled to 30 (reads are drawn at random where coverage is greater than 30). This was done to improve comparability between cases and controls and to speed up computation. A minimum QUAL of 30 was used as the threshold for calls. The GATK VQSR approach was used to filter variants across all samples. Variants with QUAL<30 and VQSLOD score equivalent to truth< = 99.9 were excluded (VQSLOD score<2.2989). Only single nucleotide polymorphism variants (SNPs) were called. There was >99% genotype concordance with genotype array data (Illumina 660k) for ∼5000 exonic SNPs with MAF>5% in the 100 resistant smokers.

### Association analyses

Single-variant association testing was undertaken using the Fisher's exact test for a comparison of resistant smoker cases and primary controls. A secondary comparison of the resistant smoker cases and the secondary controls was also undertaken although results were interpreted with caution due to disparity of the exome coverage at the pre-sequencing enrichment stage between the cases and secondary controls. Two approaches to analyse the effect of multiple variants within genes were used: SKAT (v0.92) [23] and AMELIA [24]. Variants were assigned to RefSeq genes using Annovar [53]: a total of 16439 genes were identified as containing variants in the resistant smoker cases vs. primary controls analysis. Analysis with SKAT was undertaken using default weighting to account for the assumption that rare variants are likely to have bigger effect sizes.

An alternative method, AMELIA [24], was run using a subset of the variants with MAF<5%. A total of 18182 genes were identified as containing variants (with MAF<5%), of which 7516 contained more than 4 variants and so could be reliably tested by AMELIA.

For both SKAT and AMELIA, only variants which were annotated as exonic, 5′UTR or 3′UTR were included.

### Power calculations

Power estimates for the identification of novel putatively functional variants in cases only, single-variant association tests and SKAT analysis were undertaken.

For a given variant unique to, and with a minor allele frequency of 0.005 in, resistant smokers, the probability of identifying at least one copy of the minor allele in 100 such individuals is 0.63 (0.86 for a minor allele frequency of 0.01).

Estimates of power for the single-variant association tests were undertaken for a sample size of 100 cases (resistant smokers) and 166 controls assuming a prevalence of the resistant smoker phenotype of 2% in the controls. Power calculations for detecting single variants were undertaken using Quanto and are shown in Figure S3. As an example, power to detect a variant with an allele frequency of 0.01 and an OR of 10 would be 10% for an alpha level of 5×10^−8^, and 81% for an alpha level of 0.001.

SKAT power calculations were run using the R package SKAT. The simulated dataset that the R package provides based on the coalescent populations genetic model was used to assess LD and MAF. The “Log” option was used to specify that the logOR distribution varies with allele frequency (logOR increases as minor allele frequency decreases), the effect size of each variant is equal to *c*|*log*10(MAF)|, where *c* is estimated assuming that the maximum OR corresponds to a MAF of 10^−4^. It was assumed that no logOR for causal variants was negative (results were broadly consistent if 20% of the causal variants were assumed to have negative logOR, results not shown). One thousand simulations were run for a region length of 17.7 kb (median of all gene lengths analysed in the real data), maximum OR of all variants analysed ranged from 5 to 10, significance (alpha) thresholds of 2.8×10^−6^ (Bonferroni correction for testing of 18,000 genes) and 0.01 (nominal significance threshold used to define genes as input to DAVID Gene Ontology analysis) were used and the percentage of causal variants with MAF<1% (only variants with MAF<1% were considered causal) given were 25% and 50%.

Power to detect a region of length 17.7 kb with a maximum OR of 10, assuming that 50% of variants with MAF<1% are causal is 53% for a Bonferroni-corrected significance threshold of 2.8×10^−6^, and 89% for a nominal significance threshold of 0.01 (Figure S4).

### Functional annotation and pathway enrichment analyses

We tested for enrichment of Gene Ontology terms and enrichment of signals in known biological pathways within the results of the single-variant, gene-based and case-only analyses.

A total of 150 genes had P<0.01 in the SKAT analyses (of these, 28 also had P<0.01 in the AMELIA analysis but many genes were not analysed using both SKAT and AMELIA and so only SKAT results, which included all SNPs with no MAF cut-off, were included in this analysis). A total of 1533 genes contained novel putatively functional variants in the resistant smoker cases. We tested for enrichment of Gene Ontology categories within each of these gene lists using DAVID [14] with an EASE (modified Fisher's Exact) P<0.05.

We tested for pathway enrichment within the single-variant association results using MAGENTA v2 [22]. Briefly, MAGENTA tests for deviation from a random distribution of strengths of association signals (P values) for each pathway and includes all available exome-wide single-variant association results (n = 94,138). Six databases of biological pathways were tested: including Ingenuity Pathway (June 2008, number of pathways n = 92), KEGG (2010, n = 186), PANTHER Molecular Function (January 2010, n = 276), PANTHER Biological Processes (January 2010, n = 254), PANTHER Pathways (January 2010, n = 141) and Gene Ontology (April 2010, n = 9542). Significance thresholds were Bonferroni corrected for each database.

### Immunohistochemistry

Fixed lung tissue was sectioned and mounted. Slides were treated in Histo-Clear and then re-hydrated using 100% ethanol and 95% ethanol washes. Antigen retrieval was carried out by steaming the tissue samples for 30 minutes in sodium citrate buffer (2.1 g Citric Acid [Fisons - C-6200-53]+13 ml 2M NaOH [Fisher - S-4880/53] in 87 ml H_2_O). Tissue was then treated with peroxidise blocking solution (Dako - S2023), followed by treatments with a 1 in 50 dilution of rabbit anti-CCDC38 antibody (Sigma HPA039305; 0.2 mg/ml) or a 1 in 50 dilution of the Rabbit IgG Isotype control (Invitrogen 10500C, diluted to 0.2 mg/ml). Secondary antibody staining and DAB treatment was carried out using the EnVision Detection Systems Peroxidase/DAB, Rabbit/Mouse kit (Dako – K5007). Tissue was then counterstained with Mayers Hematoxylin solution (Sigma – 51275) before being dehydrated using 95% ethanol and 100% ethanol washes. Slides were mounted using Vectamount (Vector Laboratories - H-5000).

### Lung eQTL analysis

The description of the lung eQTL dataset and subject demographics have been published previously [54]–[56]. Briefly, non-tumor lung tissues were collected from patients who underwent lung resection surgery at three participating sites: Laval University (Quebec City, Canada), University of Groningen (Groningen, The Netherlands), and University of British Columbia (Vancouver, Canada). Whole-genome gene expression and genotyping data were obtained from these specimens. Gene expression profiling was performed using an Affymetrix custom array (GPL10379) testing 51,627 non-control probe sets and normalized using RMA [57]. Genotyping was performed using the Illumina Human1M-Duo BeadChip array. Genotype imputation was undertaken using the 1000G reference panel. Following standard microarray and genotyping quality controls, 1111 patients were available including 409 from Laval, 363 from Groningen, and 339 from UBC. Lung eQTLs were identified to associate with mRNA expression in either cis (within 1 Mb of transcript start site) or in trans (all other eQTLs) and meeting the 10% false discovery rate (FDR) genome-wide significant threshold.

## Supporting Information

Figure S1 FEV1/FVC against % predicted FEV1 for the Gedling and Nottingham Smoker cohorts. The 100 samples selected as “resistant smokers” are indicated in red. The GOLD stage 2 thresholds for FEV1/FVC (0.7) and % predicted FEV1 (80%) are indicated.
(TIF)

Figure S2 Mean coverage per resistant smoker sample across whole exome.
(TIF)

Figure S3 Power to detect single variant associations (analysis 2 in the flowchart, Figure 1) for a range of odds ratios and for variants frequencies 0.5%, 1% and 3%.
(TIF)

Figure S4 Estimates of power to detect association of a region of length 17.7 kb in a case-control study using SKAT. Calculations based on simulations of a study consisting of 100 cases and 166 controls. Power estimates are shown on the y axis for a range of maximum ORs (5 to 10, x axis). Black bars represent the power assuming that 50% of all variants with MAF<1% are causal and the grey bars represent the power assuming 25% of all variants with MAF<1% are causal (we assume that only variants with MAF<1% are causal). Figure a) power to detect association reaching a Bonferroni-corrected significance threshold of 2.6×10^−8^ and figure b) power to detect association reaching a nominal significance threshold of 0.01.
(TIF)

Table S1 Sample characteristics. A) Characteristics of 100 resistant smoker samples. B) Characteristics of 230 secondary control samples. NB: Age, lung function and smoking behaviour information only available for up to 185 of the 230 samples.
(DOCX)

Table S2 Genes containing 3 novel variants predicted to be putatively functional (no gene contained more than 3 novel putatively functional variants).
(DOCX)

Table S3 Novel variants predicted to be putatively functional and identified in 2 or more samples. The final column gives the total number of novel variants predicted to be putatively functional identified in this study in each gene. REF = b37 reference allele, ALT = non-reference allele. HET = number of heterozygote individuals. ALT HOM = number of individuals homozygous for the ALT allele. ALT COUNT = total ALT allele count.
(DOCX)

Table S4 Gene Ontology (GO) terms reaching Bonferroni corrected significance for enrichment amongst the 1533 genes harbouring novel putatively functional variants in the resistant smokers, using DAVID. Ontologies: MF: Molecular Function, BP: Biological Process, CC: Cellular Component.
(DOCX)

Table S5 Lung eQTL results meeting 10% FDR for rs10859974 (chr12, non-synonymous SNP in *CCDC38*) proxy SNPs (r^2^>0.3). Z.laval, Z.Groningen and Z.UBC are the per-study estimates which were then meta-analysed. MAF: minor allele frequency. All SNPs are on chromosome 12. *r^2^ with rs10859974. ^#^First allele is eQTL coded/effect allele. (s) = synonymous, (i) = intronic. See methods.
(DOCX)

Table S6 Single-variant association results using the secondary controls for the 20 exonic SNPs showing the strongest association in the resistant smoker cases vs. Edinburgh MR-psychosis control set. NA: SNP not measured in secondary controls.
(DOCX)

Table S7 Single variant association results for SNPs within 500 kb of rs1051730 (highlighted in bold) in the 15q25.1 region which has previously shown strong association with smoking behaviour.
(DOCX)

Table S8 SKAT and AMELIA analysis results using secondary controls, ranked by SKAT P value. #Only genes with >4 SNPs with MAF<5% were tested by AMELIA. The P values for the SKAT and AMELIA analyses when Edinburgh MR-psychosis controls were used are shown in the last 2 columns. Genomic control inflation factor was 1 for SKAT and 0.99 for AMELIA.
(DOCX)
