# Supplementary figures and images for: Whole Exome Re-Sequencing Implicates CCDC38 and Cilia Structure and Function in Resistance to Smoking Related Airflow Obstruction
Source: PLoS Genet. 2014 May 1;10(5):e1004314. doi: 10.1371/journal.pgen.1004314 (PMC4006731; doi:10.1371/journal.pgen.1004314)

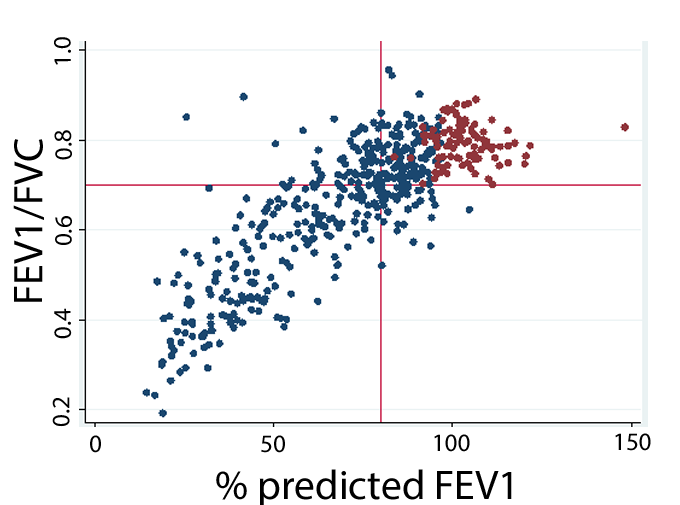

Supplement: Figure S1 — FEV1/FVC against % predicted FEV1 for the Gedling and Nottingham Smoker cohorts. The 100 samples selected as “resistant smokers” are indicated in red. The GOLD stage 2 thresholds for FEV1/FVC (0.7) and % predicted FEV1 (80%) are indicated. (TIF) [file pgen.1004314.s001.tif]

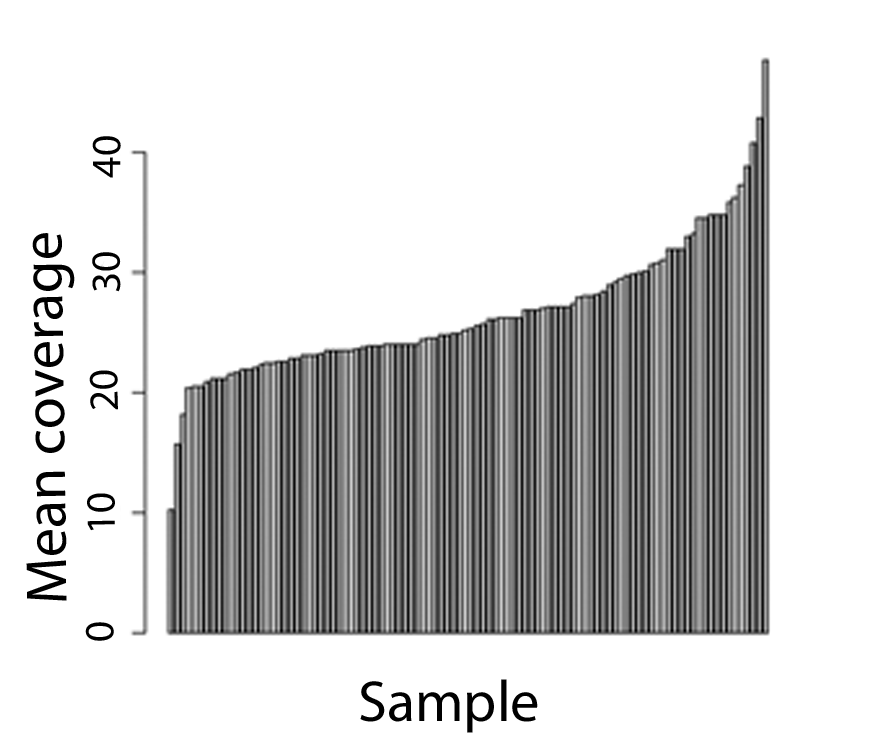

Supplement: Figure S2 — Mean coverage per resistant smoker sample across whole exome. (TIF) [file pgen.1004314.s002.tif]

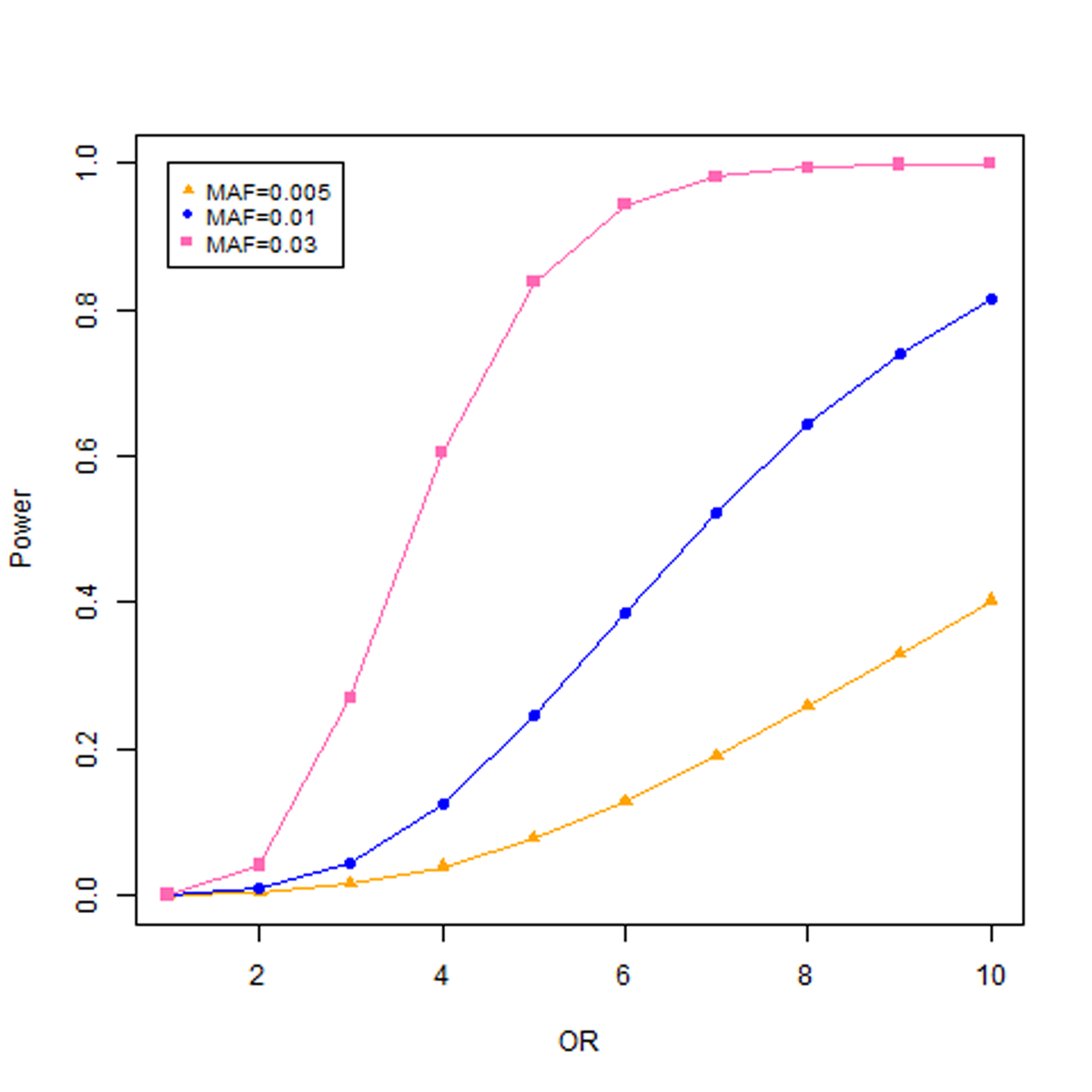

Supplement: Figure S3 — Power to detect single variant associations (analysis 2 in the flowchart, Figure 1) for a range of odds ratios and for variants frequencies 0.5%, 1% and 3%. (TIF) [file pgen.1004314.s003.tif]

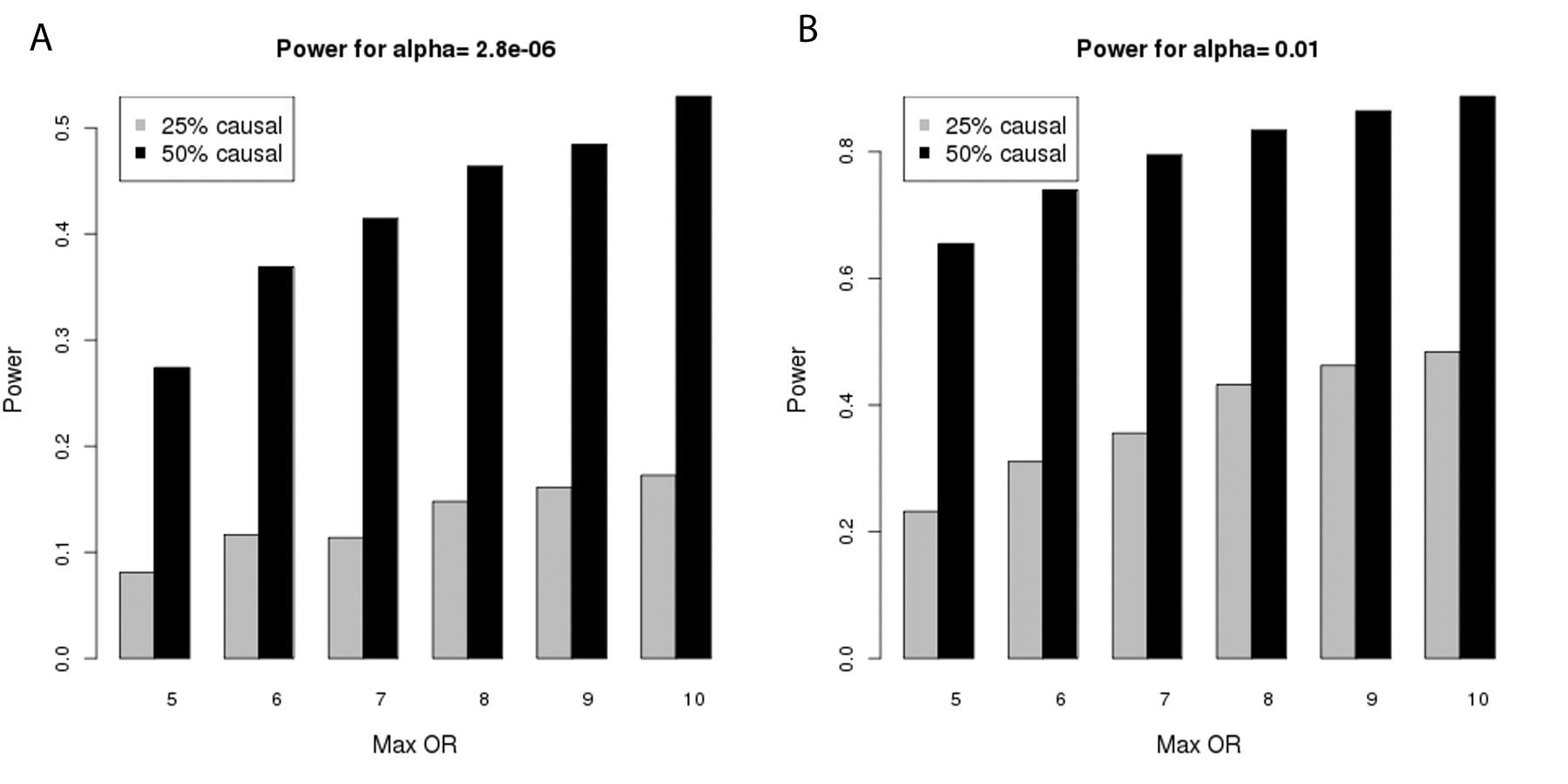

Supplement: Figure S4 — Estimates of power to detect association of a region of length 17.7 kb in a case-control study using SKAT. Calculations based on simulations of a study consisting of 100 cases and 166 controls. Power estimates are shown on the y axis for a range of maximum ORs (5 to 10, x axis). Black bars represent the power assuming that 50% of all variants with MAF<1% are causal and the grey bars represent the power assuming 25% of all variants with MAF<1% are causal (we assume that only variants with MAF<1% are causal). Figure a) power to detect association reaching a Bonferroni-corrected significance threshold of 2.6×10^−8^ and figure b) power to detect association reaching a nominal significance threshold of 0.01. (TIF) [file pgen.1004314.s004.tif]
