# Supplementary material for: Whole Exome Re-Sequencing Implicates CCDC38 and Cilia Structure and Function in Resistance to Smoking Related Airflow Obstruction
Source: PLoS Genet. 2014 May 1;10(5):e1004314. doi: 10.1371/journal.pgen.1004314 (PMC4006731; doi:10.1371/journal.pgen.1004314)
Supplement: Table S1 — Sample characteristics. A) Characteristics of 100 resistant smoker samples. B) Characteristics of 230 secondary control samples. NB: Age, lung function and smoking behaviour information only available for up to 185 of the 230 samples. (DOCX) [file pgen.1004314.s005.docx]

1. Characteristics of 100 resistant smoker samples.

| Variable | Mean | Std. Dev. | Min | Max |
| --- | --- | --- | --- | --- |
| age | 56.4 | 9.4 | 41 | 78.2 |
| FEV_1_ (l) | 3.3 | 0.6 | 1.7 | 4.7 |
| FEV_1_ (%Pred) | 103.3 | 8.8 | 83.6 | 148.2 |
| FVC (l) | 4.1 | 0.8 | 1.9 | 5.7 |
| FEV_1_/FVC | 0.79 | 0.04 | 0.70 | 0.89 |
| Pack years of smoking | 39.5 | 17.6 | 20 | 97 |
| Smoking duration (years) | 35.1 | 9.8 | 16 | 63 |
| Cigarettes per day | 24.2 | 12.8 | 5 | 80 |

Sex: 64 males, 36 females.

1. Characteristics of 230 secondary control samples. NB: Age, lung function and smoking behaviour information only available for up to 185 of the 230 samples.

| Variable | Mean | Std. Dev. | Min | Max |
| --- | --- | --- | --- | --- |
| age | 56.5 | 11 | 25 | 83 |
| FEV_1_ (l) | 2.6 | 0.5 | 1.5 | 3.9 |
| FEV_1_ (%Pred) | 106.5 | 16.3 | 55.3 | 162.5 |
| FVC (l) | 3.3 | 0.5 | 1.9 | 4.6 |
| FEV_1_/FVC | 0.8 | 0.06 | 0.62 | 1 |
| Sex: 230 females, 0 males | | | |  |
| Smoking status: | | n |  |  |
| Never smoked | | 103 |  |  |
| Current smoker | | 14 |  |  |
| Ex-smoker | | 60 |  |  |
