# Supplementary material for: Whole Exome Re-Sequencing Implicates CCDC38 and Cilia Structure and Function in Resistance to Smoking Related Airflow Obstruction
Source: PLoS Genet. 2014 May 1;10(5):e1004314. doi: 10.1371/journal.pgen.1004314 (PMC4006731; doi:10.1371/journal.pgen.1004314)
Supplement: Table S2 — Genes containing 3 novel variants predicted to be putatively functional (no gene contained more than 3 novel putatively functional variants). (DOCX) [file pgen.1004314.s006.docx]

| **Gene** | **Description** | **Length (kb)** |
| --- | --- | --- |
| *ABCA13* | ATP-binding cassette, sub-family A (ABC1), member 13 | 474.0 |
| *CLTCL1* | clathrin, heavy chain-like 1 | 111.4 |
| *CDCA7L* | cell division cycle-associated 7-like | 45.0 |
| *COG8* | conserved oligomeric Golgi complex subunit 8 | 11.0 |
| *FRMPD1* | FERM and PDZ domain containing 1 | 54.1 |
| *GBF1* | golgi brefeldin A resistant guanine nucleotide exchange factor 1 | 123.4 |
| *HIVEP1* | human immunodeficiency virus type I enhancer binding protein 1 | 148.8 |
| *LYST* | lysosomal trafficking regulator | 167.5 |
| *NAV1* | neuron navigator 1 | 171.3 |
| *NDST2* | N-deacetylase/N-sulfotransferase (heparan glucosaminyl) 2 | 9.9 |
| *NETO2* | neuropilin (NRP) and tolloid (TLL)-like 2 | 60.4 |
| *PTBP3* | polypyrimidine tract binding protein 3 | 109.6 |
| *RP1* | retinitis pigmentosa 1 (autosomal dominant) | 9.4 |
| *WFIKKN2* | WAP, follistatin/kazal, immunoglobulin, kunitz and netrin domain containing 2 | 5.1 |
| *ZBTB39* | zinc finger and BTB domain containing 39 | 2.1 |
| *ZNF789* | zinc finger protein 789 | 11.0 |
