# Supplementary material for: Whole Exome Re-Sequencing Implicates CCDC38 and Cilia Structure and Function in Resistance to Smoking Related Airflow Obstruction
Source: PLoS Genet. 2014 May 1;10(5):e1004314. doi: 10.1371/journal.pgen.1004314 (PMC4006731; doi:10.1371/journal.pgen.1004314)
Supplement: Table S3 — Novel variants predicted to be putatively functional and identified in 2 or more samples. The final column gives the total number of novel variants predicted to be putatively functional identified in this study in each gene. REF = b37 reference allele, ALT = non-reference allele. HET = number of heterozygote individuals. ALT HOM = number of individuals homozygous for the ALT allele. ALT COUNT = total ALT allele count. (DOCX) [file pgen.1004314.s007.docx]

| **Gene** | **Variant (chr:pos) b37/hg19** | **REF** | **ALT** | **HET** | **ALT HOM** | **Hardy Weinberg Disequilibrium P value** | **Total # putatively functional variants in gene** |
| --- | --- | --- | --- | --- | --- | --- | --- |
| *ATAD3C* | 1:1392552 | C | G | 6 | 0 | 0.1 | 1 |
| *SHANK2* | 11:70794454 | C | T | 3 | 0 | 0.4 | 1 |
| *ARGFX* | 3:121289580 | C | T | 2 | 0 | 1 | 1 |
| *MED11* | 17:4635131 | C | G | 2 | 0 | 1 | 1 |
| *GBF1* | 10:104117872 | A | G | 2 | 0 | 1 | 3 |
| *MUC3A* | 7:100550320 | A | C | 2 | 0 | 1 | 1 |
| *SPATA20* | 17:48627593 | C | T | 2 | 0 | 1 | 1 |
| *TSHZ1* | 18:72998971 | C | T | 2 | 0 | 1 | 2 |
