# Supplementary material for: Whole Exome Re-Sequencing Implicates CCDC38 and Cilia Structure and Function in Resistance to Smoking Related Airflow Obstruction
Source: PLoS Genet. 2014 May 1;10(5):e1004314. doi: 10.1371/journal.pgen.1004314 (PMC4006731; doi:10.1371/journal.pgen.1004314)
Supplement: Table S4 — Gene Ontology (GO) terms reaching Bonferroni corrected significance for enrichment amongst the 1533 genes harbouring novel putatively functional variants in the resistant smokers, using DAVID. Ontologies: MF: Molecular Function, BP: Biological Process, CC: Cellular Component. (DOCX) [file pgen.1004314.s008.docx]

| GO ontology: accession~term | GO definition | # genes overlapping GO term | % of total genes tested overlapping GO term | DAVID EASE P Value | Bonferroni corrected P value |
| --- | --- | --- | --- | --- | --- |
| MF:GO:0003774~motor activity | Catalysis of movement along a polymeric molecule such as a microfilament or microtubule, coupled to the hydrolysis of a nucleoside triphosphate. | 39 | 2.5 | 1.78x10^-10^ | 2.08x10^-7^ |
| BP:GO:0007155~cell adhesion | The attachment of a cell, either to another cell or to an underlying substrate such as the extracellular matrix, via cell adhesion molecules. | 105 | 6.8 | 3.01x10^-8^ | 1.02x10^-4^ |
| BP:GO:0022610~biological adhesion | The attachment of a cell or organism to a substrate or other organism. | 105 | 6.8 | 3.22x10^-8^ | 1.09x10^-4^ |
| CC:GO:0005856~cytoskeleton | Any of the various filamentous elements that form the internal framework of cells, and typically remain after treatment of the cells with mild detergent to remove membrane constituents and soluble components of the cytoplasm. The term embraces intermediate filaments, microfilaments, microtubules, the microtrabecular lattice, and other structures characterized by a polymeric filamentous nature and long-range order within the cell. The various elements of the cytoskeleton not only serve in the maintenance of cellular shape but also have roles in other cellular functions, including cellular movement, cell division, endocytosis, and movement of organelles. | 169 | 11.0 | 4.30x10^-8^ | 2.49x10^-5^ |
| MF:GO:0030554~adenyl nucleotide binding | Interacting selectively and non-covalently with adenyl nucleotides, any compound consisting of adenosine esterified with (ortho)phosphate. | 191 | 12.5 | 7.36x10^-7^ | 8.59x10^-4^ |
| MF:GO:0005509~calcium ion binding | Interacting selectively and non-covalently with calcium ions (Ca2+). | 123 | 8.0 | 8.65x10^-7^ | 1.01x10^-3^ |
| MF:GO:0016887~ATPase activity | Catalysis of the reaction: ATP + H2O = ADP + phosphate + 2 H+. May or may not be coupled to another reaction. | 57 | 3.7 | 1.14x10^-6^ | 1.33x10^-3^ |
| MF:GO:0001883~purine nucleoside binding | Interacting selectively and non-covalently with a purine nucleoside, a compound consisting of a purine base linked either to ribose or deoxyribose. | 191 | 12.5 | 2.08x10^-6^ | 2.43x10^-3^ |
| MF:GO:0001882~nucleoside binding | Interacting selectively and non-covalently with a nucleoside, a compound consisting of a purine or pyrimidine nitrogenous base linked either to ribose or deoxyribose. | 192 | 12.5 | 2.13x10^-6^ | 2.48x10^-3^ |
| CC:GO:0005930~axoneme | The bundle of microtubules and associated proteins that forms the core of cilia and flagella in eukaryotic cells and is responsible for their movements. | 15 | 1.0 | 2.44x10^-6^ | 1.41x10^-3^ |
| MF:GO:0005524~ATP binding | Interacting selectively and non-covalently with ATP, adenosine 5'-triphosphate, a universally important coenzyme and enzyme regulator. | 178 | 11.6 | 2.73x10^-6^ | 3.18x10^-3^ |
| CC:GO:0016459~myosin complex | A protein complex, formed of one or more myosin heavy chains plus associated light chains and other proteins, that functions as a molecular motor; uses the energy of ATP hydrolysis to move actin filaments or to move vesicles or other cargo on fixed actin filaments; has magnesium-ATPase activity and binds actin. Myosin classes are distinguished based on sequence features of the motor, or head, domain, but also have distinct tail regions that are believed to bind specific cargoes. | 19 | 1.2 | 2.95x10^-6^ | 1.71x10^-3^ |
| MF:GO:0003777~microtubule motor activity | Catalysis of movement along a microtubule, coupled to the hydrolysis of a nucleoside triphosphate (usually ATP). | 21 | 1.4 | 6.05x10^-6^ | 7.04x10^-3^ |
| MF:GO:0032559~adenyl ribonucleotide binding | Interacting selectively and non-covalently with an adenyl ribonucleotide, any compound consisting of adenosine esterified with (ortho)phosphate or an oligophosphate at any hydroxyl group on the ribose moiety. | 178 | 11.6 | 6.21x10^-6^ | 7.23x10^-3^ |
| CC:GO:0031012~extracellular matrix | A structure lying external to one or more cells, which provides structural support for cells or tissues; may be completely external to the cell (as in animals and bacteria) or be part of the cell (as in plants). | 53 | 3.5 | 1.22x10^-5^ | 7.06x10^-3^ |
| CC:GO:0005929~cilium | A specialized eukaryotic organelle that consists of a filiform extrusion of the cell surface. Each cilium is bounded by an extrusion of the cytoplasmic membrane, and contains a regular longitudinal array of microtubules, anchored basally in a centriole. | 27 | 1.8 | 1.41x10^-5^ | 8.14x10^-3^ |
| CC:GO:0005578~proteinaceous extracellular matrix | A layer consisting mainly of proteins (especially collagen) and glycosaminoglycans (mostly as proteoglycans) that forms a sheet underlying or overlying cells such as endothelial and epithelial cells. The proteins are secreted by cells in the vicinity. | 50 | 3.3 | 1.41x10^-5^ | 8.15x10^-3^ |
| MF:GO:0046872~metal ion binding | Interacting selectively and non-covalently with any metal ion. | 423 | 27.6 | 1.97x10^-5^ | 2.28x10^-2^ |
| CC:GO:0044420~extracellular matrix part | Any constituent part of the extracellular matrix, the structure lying external to one or more cells, which provides structural support for cells or tissues; may be completely external to the cell (as in animals) or be part of the cell (as often seen in plants). | 25 | 1.6 | 2.20x10^-5^ | 1.27x10^-2^ |
| MF:GO:0043169~cation binding | Interacting selectively and non-covalently with cations, charged atoms or groups of atoms with a net positive charge. | 426 | 27.8 | 2.32x10^-5^ | 2.67x10^-2^ |
| MF:GO:0043167~ion binding | Interacting selectively and non-covalently with ions, charged atoms or groups of atoms. | 431 | 28.1 | 2.74x10^-5^ | 3.15x10^-2^ |
| CC:GO:0044441~cilium part | Any constituent part of a cilium, a specialized eukaryotic organelle that consists of a filiform extrusion of the cell surface. Each cilium is bounded by an extrusion of the cytoplasmic membrane, and contains a regular longitudinal array of microtubules, anchored basally in a centriole. | 14 | 0.9 | 3.29x10^-5^ | 1.89x10^-2^ |
