# Supplementary material for: Whole Exome Re-Sequencing Implicates CCDC38 and Cilia Structure and Function in Resistance to Smoking Related Airflow Obstruction
Source: PLoS Genet. 2014 May 1;10(5):e1004314. doi: 10.1371/journal.pgen.1004314 (PMC4006731; doi:10.1371/journal.pgen.1004314)
Supplement: Table S5 — Lung eQTL results meeting 10% FDR for rs10859974 (chr12, non-synonymous SNP in CCDC38) proxy SNPs (r2>0.3). Z.laval, Z.Groningen and Z.UBC are the per-study estimates which were then meta-analysed. MAF: minor allele frequency. All SNPs are on chromosome 12. *r2 with rs10859974. #First allele is eQTL coded/effect allele. (s) = synonymous, (i) = intronic. See methods. (DOCX) [file pgen.1004314.s009.docx]

| Proxy SNP | Position (hg19) | r^2*^ | SNP location | eQTL P | Z Laval | Z Groningen | Z UBC | eQTL regulated gene | Alleles^#^ | MAF |
| --- | --- | --- | --- | --- | --- | --- | --- | --- | --- | --- |
| rs11108320 | 96288585 | 1 | CCDC38 (i) | 1.52E-05 | -3.669 | -1.468 | -2.273 | NTN4 | A G | 0.172 |
| rs77173765 | 96285140 | 0.925 | N/A | 4.84E-06 | -3.716 | -1.569 | -2.576 | NTN4 | T G | 0.172 |
| rs17024721 | 96203046 | 0.778 | N/A | 1.37E-06 | -3.772 | -1.460 | -3.185 | NTN4 | A T | 0.163 |
| rs976406 | 96227439 | 0.778 | N/A | 2.20E-06 | -3.724 | -1.447 | -3.070 | NTN4 | T G | 0.167 |
| rs17024778 | 96230404 | 0.778 | N/A | 1.37E-06 | -3.772 | -1.460 | -3.186 | NTN4 | G A | 0.163 |
| rs11108299 | 96238254 | 0.778 | N/A | 1.29E-06 | -3.772 | -1.460 | -3.213 | NTN4 | C T | 0.162 |
| rs73371127 | 96239511 | 0.778 | N/A | 1.29E-06 | -3.772 | -1.460 | -3.213 | NTN4 | C A | 0.162 |
| rs10859962 | 96243641 | 0.778 | N/A | 1.48E-06 | -3.768 | -1.486 | -3.144 | NTN4 | A T | 0.160 |
| rs10859963 | 96245366 | 0.778 | N/A | 1.29E-06 | -3.772 | -1.461 | -3.213 | NTN4 | G A | 0.162 |
| rs12579712 | 96246313 | 0.778 | N/A | 1.20E-06 | -3.772 | -1.484 | -3.213 | NTN4 | C T | 0.162 |
| rs12370255 | 96248540 | 0.778 | N/A | 1.13E-06 | -3.758 | -1.544 | -3.184 | NTN4 | A C | 0.162 |
| rs12579279 | 96250125 | 0.778 | N/A | 9.58E-07 | -3.772 | -1.571 | -3.205 | NTN4 | G T | 0.161 |
| rs11108307 | 96250582 | 0.778 | N/A | 9.81E-07 | -3.777 | -1.573 | -3.188 | NTN4 | A G | 0.161 |
| rs17024787 | 96254880 | 0.778 | SNRPF (i) | 8.23E-07 | -3.851 | -1.666 | -3.026 | NTN4 | C T | 0.162 |
| rs28762087 | 96256261 | 0.778 | SNRPF (i) | 9.87E-07 | -4.042 | -1.311 | -3.204 | NTN4 | G A | 0.171 |
| rs12582750 | 96265521 | 0.778 | CCDC38 (i) | 3.52E-07 | -3.922 | -1.873 | -3.025 | NTN4 | G T | 0.162 |
| rs2117914 | 96266035 | 0.778 | CCDC38(s) | 3.52E-07 | -3.922 | -1.873 | -3.025 | NTN4 | T C | 0.162 |
| rs17024820 | 96268393 | 0.778 | CCDC38 (i) | 3.52E-07 | -3.922 | -1.873 | -3.025 | NTN4 | A C | 0.162 |
| rs10507065 | 96269399 | 0.778 | CCDC38 (i) | 3.48E-07 | -3.923 | -1.875 | -3.025 | NTN4 | A G | 0.162 |
| rs11108314 | 96273122 | 0.778 | CCDC38 (i) | 1.31E-07 | -3.928 | -2.148 | -3.046 | NTN4 | C T | 0.160 |
| rs10859970 | 96277265 | 0.778 | CCDC38 (i) | 3.51E-07 | -3.922 | -1.873 | -3.025 | NTN4 | T C | 0.162 |
| rs10859971 | 96277785 | 0.778 | CCDC38 (i) | 3.49E-07 | -3.923 | -1.876 | -3.022 | NTN4 | T C | 0.162 |
| rs11108317 | 96277930 | 0.778 | CCDC38 (i) | 3.49E-07 | -3.923 | -1.877 | -3.021 | NTN4 | T C | 0.162 |
| rs11108319 | 96279180 | 0.778 | CCDC38 (i) | 3.23E-07 | -3.924 | -1.904 | -3.014 | NTN4 | C G | 0.162 |
| rs1898180 | 96281140 | 0.778 | CCDC38 (i) | 5.56E-07 | -3.893 | -1.815 | -2.990 | NTN4 | C T | 0.163 |
| rs1898179 | 96281172 | 0.778 | CCDC38 (i) | 5.53E-07 | -3.888 | -1.815 | -3.006 | NTN4 | G A | 0.163 |
| rs11108272 | 96196005 | 0.714 | N/A | 3.89E-08 | -4.070 | -1.996 | -3.570 | NTN4 | T C | 0.174 |
| rs10859958 | 96212532 | 0.714 | N/A | 1.21E-06 | -4.062 | -1.106 | -3.360 | NTN4 | C T | 0.174 |
| rs58629661 | 96231501 | 0.714 | N/A | 1.35E-06 | -3.772 | -1.460 | -3.193 | NTN4 | C A | 0.163 |
| rs11108326 | 96299314 | 0.714 | CCDC38 (i) | 5.59E-07 | -3.407 | -2.592 | -2.704 | NTN4 | T C | 0.221 |
| rs1436118 | 96220112 | 0.704 | N/A | 1.37E-06 | -3.771 | -1.465 | -3.180 | NTN4 | T C | 0.163 |
| rs11108298 | 96231927 | 0.704 | N/A | 1.34E-06 | -3.772 | -1.460 | -3.197 | NTN4 | C T | 0.163 |
| rs11836265 | 96283908 | 0.659 | CCDC38 (i) | 4.27E-07 | -3.901 | -1.498 | -3.434 | NTN4 | T G | 0.174 |
| rs7135865 | 96290173 | 0.64 | CCDC38 (i) | 1.92E-07 | -3.749 | -2.326 | -3.052 | NTN4 | A T | 0.228 |
| rs11108271 | 96194399 | 0.346 | N/A | 2.33E-10 | -5.211 | -1.452 | -4.211 | NTN4 | T C | 0.286 |
| rs12582105 | 96198627 | 0.346 | N/A | 2.32E-10 | -5.211 | -1.458 | -4.209 | NTN4 | G A | 0.286 |
| rs73369383 | 96198898 | 0.346 | N/A | 1.96E-10 | -5.226 | -1.484 | -4.210 | NTN4 | C G | 0.286 |
| rs10859956 | 96204979 | 0.346 | N/A | 2.32E-10 | -5.210 | -1.460 | -4.208 | NTN4 | T C | 0.286 |
| rs7307769 | 96205888 | 0.346 | N/A | 9.83E-10 | -5.014 | -1.445 | -4.042 | NTN4 | G A | 0.292 |
| rs4762627 | 96209174 | 0.346 | N/A | 2.50E-10 | -5.195 | -1.460 | -4.208 | NTN4 | G A | 0.286 |
| rs7134181 | 96214200 | 0.346 | N/A | 1.40E-09 | -4.950 | -1.251 | -4.218 | NTN4 | C T | 0.288 |
| rs10859959 | 96216680 | 0.346 | N/A | 2.80E-10 | -5.167 | -1.460 | -4.208 | NTN4 | T C | 0.286 |
| rs10859960 | 96218158 | 0.346 | N/A | 2.80E-10 | -5.167 | -1.460 | -4.208 | NTN4 | G A | 0.286 |
| rs1972293 | 96221800 | 0.346 | N/A | 2.80E-10 | -5.167 | -1.459 | -4.208 | NTN4 | A G | 0.286 |
| rs7135910 | 96289948 | 0.333 | CCDC38 (i) | 5.11E-06 | 3.521 | 1.170 | 3.323 | SNRPF | G A | 0.356 |
| rs964127 | 96290733 | 0.333 | CCDC38 (i) | 5.71E-06 | 3.514 | 1.165 | 3.287 | SNRPF | A T | 0.356 |
| rs1369825 | 96291697 | 0.333 | CCDC38 (i) | 7.14E-06 | 3.505 | 1.117 | 3.296 | SNRPF | C T | 0.355 |
| rs12366571 | 96214566 | 0.329 | N/A | 3.72E-12 | -5.448 | -1.970 | -4.598 | NTN4 | A C | 0.297 |
| rs79932769 | 96245841 | 0.304 | N/A | 1.72E-05 | -2.454 | -1.369 | -4.048 | NTN4 | A G | 0.067 |
