# Supplementary material for: Whole Exome Re-Sequencing Implicates CCDC38 and Cilia Structure and Function in Resistance to Smoking Related Airflow Obstruction
Source: PLoS Genet. 2014 May 1;10(5):e1004314. doi: 10.1371/journal.pgen.1004314 (PMC4006731; doi:10.1371/journal.pgen.1004314)
Supplement: Table S6 — Single-variant association results using the secondary controls for the 20 exonic SNPs showing the strongest association in the resistant smoker cases vs. Edinburgh MR-psychosis control set. NA: SNP not measured in secondary controls. (DOCX) [file pgen.1004314.s010.docx]

| SNP | CHR | BP | A1 | A2 | Case MAF | 2^o^ controls MAF | snp_type | gene | hg19.kgXref.description | Fisher P |
| --- | --- | --- | --- | --- | --- | --- | --- | --- | --- | --- |
| rs1287467 | 3 | 15311325 | A | G | 0.235 | 0.296 | Synonymous | *SH3BP5* | SH3-domain binding protein 5 (BTK-associated) | 1.30x10-^1^ |
| rs2303296 | 2 | 24432839 | G | A | 0.170 | 0.274 | Synonymous | *ITSN2* | intersectin 2 | 4.12x10^-3^ |
| rs10859974 | 12 | 96288860 | C | T | 0.255 | 0.157 | nonsynonymous | *CCDC38* | coiled-coil domain containing 38 (CCDC38), mRNA. | 4.51x10^-3^ |
| rs4850 | 16 | 21976762 | A | G | 0.095 | 0.065 | nonsynonymous | *UQCRC2* | ubiquinol-cytochrome c reductase core protein II, nuclear gene encoding mitochondrial protein | 1.97x10^-1^ |
| rs1566290 | 17 | 71239087 | T | G | 0.295 | NA | Synonymous | *C17orf80* | chromosome 17 open reading frame 80 | NA |
| rs35853276 | 2 | 174055646 | C | T | 0.040 | 0.107 | Synonymous | *ZAK* | sterile alpha motif and leucine zipper containing kinase AZK | 4.06x10^-3^ |
| rs13184586 | 5 | 161119125 | C | G | 0.485 | 0.413 | Synonymous | *GABRA6* | gamma-aminobutyric acid (GABA) A receptor, alpha 6 | 8.84x10^-2^ |
| rs2297950 | 1 | 203194186 | T | C | 0.250 | NA | nonsynonymous | *CHIT1* | chitinase 1 (chitotriosidase) | NA |
| rs7709828 | 5 | 94786142 | T | C | 0.035 | NA | UTR3 | *FAM81B* | family with sequence similarity 81, member B | NA |
| rs1046515 | 7 | 140394587 | T | C | 0.030 | NA | nonsynonymous | *ADCK2* | aarF domain containing kinase 2 | NA |
| rs17010021 | 2 | 74761539 | A | T | 0.000 | 0.061 | nonsynonymous | *LOXL3* | lysyl oxidase-like 3 | 6.27x10^-5^ |
| rs2878 | 7 | 102953621 | G | A | 0.060 | NA | UTR3 | *PMPCB* | peptidase (mitochondrial processing) beta, nuclear gene encoding mitochondrial protein, | NA |
| rs34350265 | 9 | 116973273 | T | C | 0.115 | NA | Synonymous | *COL27A1* | collagen, type XXVII, alpha 1 | NA |
| rs111336032 | 17 | 80017898 | A | G | 0.045 | NA | nonsynonymous | *DUS1L* | dihydrouridine synthase 1-like (S. cerevisiae) | NA |
| rs6979 | 16 | 67691668 | G | A | 0.575 | NA | nonsynonymous | *ACD* | adrenocortical dysplasia homolog (mouse) | NA |
| rs2295879 | 10 | 123996976 | A | G | 0.395 | 0.315 | nonsynonymous | *TACC2* | transforming, acidic coiled-coil containing protein 2 | 7.32x10^-2^ |
| rs2427808 | 1 | 158577167 | T | A | 0.170 | 0.130 | Synonymous | *OR10Z1* | olfactory receptor, family 10, subfamily Z, member 1 | 1.84x10^-1^ |
| rs17680262 | 12 | 110354536 | T | C | 0.170 | NA | UTR3 | *TCHP* | trichoplein, keratin filament binding | NA |
| rs35464006 | 17 | 37840860 | C | G | 0.035 | NA | nonsynonymous | *PGAP3* | post-GPI attachment to proteins 3 | NA |
| rs116948895 | 9 | 84609249 | T | A | 0.035 | 0.009 | Synonymous | *SPATA3D1* | family with sequence similarity 75, member D1 | 2.19x10^-2^ |
