# Supplementary material for: Whole Exome Re-Sequencing Implicates CCDC38 and Cilia Structure and Function in Resistance to Smoking Related Airflow Obstruction
Source: PLoS Genet. 2014 May 1;10(5):e1004314. doi: 10.1371/journal.pgen.1004314 (PMC4006731; doi:10.1371/journal.pgen.1004314)
Supplement: Table S7 — Single variant association results for SNPs within 500 kb of rs1051730 (highlighted in bold) in the 15q25.1 region which has previously shown strong association with smoking behaviour. (DOCX) [file pgen.1004314.s011.docx]

| **SNP** | **Chr15 position** | **gene** | **A1** | **A2** | **MAF** | **Case MAF** | **1^o^ control MAF** | **1^o^ control Fisher P** | **type** | **2^o^ control MAF** | **2^o^ control Fisher P** |
| --- | --- | --- | --- | --- | --- | --- | --- | --- | --- | --- | --- |
| rs8040868 | 78911181 | *CHRNA3* | C | T | 0.377 | 0.434 | 0.343 | 0.052 | synonymous | NA | NA |
| rs61752770 | 78454050 | *IDH3A* | A | T | 0.006 | 0.015 | 0.000 | 0.053 | non-synonymous | 0.004 | 0.166 |
| rs147992380 | 79382733 | *RASGRF1* | A | G | 0.006 | 0.015 | 0.000 | 0.053 | synonymous | NA | NA |
| rs13180 | 78789488 | *IREB2* | C | T | 0.378 | 0.325 | 0.410 | 0.053 | synonymous | 0.428 | 0.015 |
| **rs1051730** | **78894339** | ***CHRNA3*** | **A** | **G** | **0.333** | **0.385** | **0.301** | **0.057** | **synonymous** | **0.287** | **0.028** |
| rs16969968 | 78882925 | *CHRNA5* | A | G | 0.332 | 0.384 | 0.301 | 0.071 | non-synonymous | 0.287 | 0.023 |
| rs894780 | 79298523 | *RASGRF1* | A | G | 0.113 | 0.145 | 0.093 | 0.089 | synonymous | NA | NA |
| rs3885951 | 78825917 | *AGPHD1* | G | A | 0.098 | 0.120 | 0.084 | 0.228 | non-synonymous | 0.057 | 0.006 |
| rs56317523 | 78921343 | *CHRNB4* | A | G | 0.006 | 0.010 | 0.003 | 0.560 | non-synonymous | 0.000 | 0.092 |
| rs116374996 | 78461324 | *IDH3A* | T | C | 0.019 | 0.020 | 0.018 | 1.000 | non-synonymous | 0.002 | 0.031 |
