# Supplementary material for: Whole Exome Re-Sequencing Implicates CCDC38 and Cilia Structure and Function in Resistance to Smoking Related Airflow Obstruction
Source: PLoS Genet. 2014 May 1;10(5):e1004314. doi: 10.1371/journal.pgen.1004314 (PMC4006731; doi:10.1371/journal.pgen.1004314)
Supplement: Table S8 — SKAT and AMELIA analysis results using secondary controls, ranked by SKAT P value. #Only genes with >4 SNPs with MAF<5% were tested by AMELIA. The P values for the SKAT and AMELIA analyses when Edinburgh MR-psychosis controls were used are shown in the last 2 columns. Genomic control inflation factor was 1 for SKAT and 0.99 for AMELIA. (DOCX) [file pgen.1004314.s012.docx]

|  | SKAT | | AMELIA | |  | Results using primary controls | |
| --- | --- | --- | --- | --- | --- | --- | --- |
| gene | Variants | SKAT P | <5% MAF | AMELIA P# | Description | SKAT P | AMELIA P |
| *RPTN* | 19 | 4.73X10^-5^ | 17 | 5.30X10^-2^ | Repetin | 2.33x10^-1^ | 3.88x10^-1^ |
| *OPN5* | 6 | 9.58X10^-5^ | 6 | 2.00X10^-6^ | opsin 5 | 3.66x10^-2^ | NA |
| *BTN2A3P* | 7 | 9.69X10^-5^ | 5 | 1.77X10^-4^ | butyrophilin, subfamily 2, member A3, pseudogene | 1 | 9.76x10^-1^ |
| *HCLS1* | 14 | 1.15X10^-4^ | 8 | 3.09X10^-1^ | hematopoietic cell-specific Lyn substrate 1 | 7.37x10^-1^ | 9.05x10^-1^ |
| *PTCH2* | 6 | 1.58X10^-4^ | 5 | 3.44X10^-4^ | patched 2 | 3.05x10^-2^ | 9.30x10^-2^ |
| *DTNA* | 6 | 1.59X10^-4^ | 6 | 3.22X10^-4^ | dystrobrevin, alpha | 4.04x10^-2^ | 2.70x10^-2^ |
| *KIAA0754* | 9 | 2.38X10^-4^ | 9 | 6.29X10^-3^ | KIAA0754 | 2.67x10^-1^ | 1.85x10^-1^ |
| *DIS3L* | 8 | 2.53X10^-4^ | 0 | - | DIS3 mitotic control homolog (S. cerevisiae)-like | 9.32x10^-1^ | 9.02x10^-1^ |
| *SNAPC3* | 2 | 3.14X10^-4^ | 0 | - | small nuclear RNA activating complex, polypeptide 3, 50kDa | 7.63x10^-2^ | NA |
| *ILK* | 8 | 3.40X10^-4^ | 5 | <10^-7^ | integrin-linked kinase | 5.68x10^-3^ | NA |
| *TMEM252* | 4 | 3.90X10^-4^ | 0 | - | chromosome 9 open reading frame 71 | 4.38x10^-4^ | NA |
| *TMEM71* | 11 | 4.10X10^-4^ | 9 | 6.19X10^-4^ | transmembrane protein 71 | 4.49x10^-1^ | NA |
| *SAMD12* | 6 | 4.41X10^-4^ | 0 | - | sterile alpha motif domain containing 12 | 3.74x10^-1^ | NA |
| *SEPW1* | 2 | 5.04X10^-4^ | 0 | - | selenoprotein W, 1 | 2.61x10^-1^ | NA |
| *DLL1* | 4 | 6.05X10^-4^ | 0 | - | delta-like 1 (Drosophila) | 2.55x10^-2^ | 2.20x10^-2^ |
| *CHRNE* | 4 | 8.40X10^-4^ | 4 | 6.02X10^-3^ | cholinergic receptor, nicotinic, epsilon | 5.35x10^-1^ | 8.70x10^-1^ |
| *VPS37C* | 2 | 8.45X10^-4^ | 0 | - | vacuolar protein sorting 37 homolog C (S. cerevisiae) | 2.50x10^-1^ | NA |
| *LRP2BP* | 6 | 1.25X10^-3^ | 5 | <10^-7^ | LRP2 binding protein | 4.41x10^-1^ | 5.20x10^-1^ |
| *SGSM2* | 5 | 4.93X10^-3^ | 5 | 7.37X10^-4^ | small G protein signalling modulator 2 | 5.70x10^-1^ | 5.24x10^-1^ |
| *DRAM2* | 5 | 1.13X10^-2^ | 5 | <10^-7^ | DNA-damage regulated autophagy modulator 2 | 3.66x10^-2^ | NA |
